# Graduate students and supervisors matching decision-making considering stability-based fairness based on TOPSIS and grey correlation degrees

**DOI:** 10.1038/s41598-025-00388-6

**Published:** 2025-07-02

**Authors:** Xiaohua Liu, Qi Yue, Bin Hu, Yuan Tao

**Affiliations:** School of Management, Shanghai University of Engineering Science, Shanghai, 201620 China

**Keywords:** Graduate students and supervisors matching decision-making, Pythagorean fuzzy environment, Conformity psychology, Stability-based fairness, Many-to-one matching, Applied mathematics, Information technology

## Abstract

In view of the lack of research on graduate students and supervisors matching (GSSM) decision-making, this study proposes a novel many-to-one matching decision-making method for graduate students and supervisors (GSS) considering conformity psychology and graduate students’ preferences from a stability-based fairness perspective. First, the many-to-one matching problem for GSS is described. To tackle this problem, linguistic term matrices (LTMs) provided by bilateral subjects are transformed into Pythagorean fuzzy matrices (PFMs). On the one hand, according to the transference relation of graduate students, a conformity coefficient matrix is built. Then a PFM considering conformity psychology is established. Attribute weight vectors adjusted by conformity coefficients are formulated based on comparison information of graduate students and the conformity coefficient matrix. On this basis, a comprehensive PFM of graduate students is constructed. On the other hand, a preference coefficient matrix of graduate students is built by using TODIM (a Portuguese acronym for interactive and multicriteria decision making). And a PFM considering graduate students’ preferences is constructed. Attribute weight vectors of supervisors are determined based on attribute comparison information. On this basis, a comprehensive PFM of supervisors is set up. Furthermore, satisfaction matrices of GSS are constructed by using TOPSIS (technique for order preference by similarity to the ideal solution) and grey correlation degrees. A many-to-one GSSM model considering stability-based fairness is established by introducing matching matrix and stable matching matrix. The many-to-one matching model is then transformed into a one-to-one matching model by introducing virtual supervisor subjects; the optimal matching scheme between GSS is obtained by solving the above model. Finally, the feasibility, effectiveness and innovation of the proposed method are verified by an example analysis. The key findings of this study are listed as follows: (1) A new score of Pythagorean fuzzy numbers (PFNs) is proposed. (2) A method for converting linguistic term sets (LTSs) into PFNs is developed. (3) A weight calculation that combines conformity psychology with BWM is improved. (4) A novel method for satisfaction calculation considering conformity psychology and graduate students’ preferences is introduced. (5) A GSSM model considering stability-based fairness is built.

## Introduction

The global higher education landscape is undergoing paradigmatic shifts, driven by evolving knowledge production paradigms. Within this context, graduate student-supervisor matching^1^ (GSSM) has emerged as a critical determinant of research ecosystem sustainability and academic equity. Although the importance of GSSM has been widely recognized, two persistent challenges demand resolution: First, at the operational level, the traditional administrative led “one-way selection” model is prone to insufficient matching stability^2^, resulting in research direction deviation between graduate students and supervisors (GSS) after matching, and teacher-student conflicts^3^ caused by matching failure. Second, at the value level, implicit preferences in the matching process may undermine fairness, and disadvantaged graduate students are systematically disadvantaged in mentor selection, with a lower probability of receiving guidance from top mentors compared to advantaged groups. Therefore, building a GSSM decision model that balances stability and fairness has urgent practical significance. This research can not only improve theoretical and methodological systems of higher education management, but also provide key decision support for the reform and innovation of graduate training mechanisms. Therefore, GSSM decision-making considering stability and fairness is a worthwhile research theme.

In real-world decision-making environments, it is generally easier for both subjects to provide linguistic evaluations rather than numerical ones for attributes. Therefore, this paper assumes that the evaluation information provided by GSS is in the form of linguistic term sets (LTSs)^4^. However, LTSs lack quantitative ability and mathematical rigor. Additionally, intuitionistic fuzzy numbers^5^ are effective in simple conflict scenarios, but they are limited by the strong constraint of $u + v \le 1$. Compared with LTSs and intuitionistic fuzzy numbers, Pythagorean fuzzy numbers (PFNs) are more suitable for practical problems with complex information. Since PFNs can more comprehensively address uncertainty, reduce errors caused by fuzzy information and enhance the scientific reliability of decision-making^6^, the method for converting LTSs into PFNs should be proposed to avoid potential information loss.

Conformity behavior is a common phenomenon in group within the socio-economic context^7^. It refers to situations where decision-makers tend to believe that other participants have access to more comprehensive information under conditions of information asymmetry and expectations uncertainty. As a result, they are more likely to align their decisions with those of others^8^. In complex decision-making scenarios, graduate students often lack sufficient confidence or judgment, leading them to rely on the choices of others. This tendency is typically attributed to the conformity psychology. Therefore, this paper considers introducing conformity psychology into graduate student preferences. Relatively speaking, the phenomenon of conformity among supervisors is not obvious. Supervisors usually make choices based on professional judgments and research interests. However, supervisors usually take into account wishes of graduate students when making decisions. Respecting the wishes of graduate students will increase trust and respect for supervisors, which is beneficial for establishing a positive student-supervisor relationship. Therefore, this paper considers incorporating graduate student preferences into the calculation of supervisor preferences.

TODIM (a Portuguese acronym for interactive and multicriteria decision making) was first proposed by Gomes and Lima^9^ in 1992 and applied in academ. Currently, TODIM has been continuously expanded and used by scholars. For example, Li et al.^10^ proposed a novel analytical model that integrates interval type-2 fuzzy sets, cumulative prospect theory and TODIM method to evaluate credit risk in supply chain finance; Zhao et al.^11^ proposed an interval-valued Pythagorean fuzzy method using the classical TODIM based on cumulative prospect theory; Arya and Kumar^12^ studied a picture fuzzy multiple criteria decision-making approach based on the improved TODIM. The TODIM method’s popularity stems from its ability to consider decision-maker’s preferences, handle incomplete and imprecise information, deal with non-compensatory and non-linear relationships, have a transparent process, and have wide applicability. Consequently, this study employs TODIM to calculate the graduate students’ preference.

Based on the above analysis, this paper focuses on proposing a many-to-one matching decision-making method for GSS from the perspective of stability-based fairness, which takes into account conformity psychology and graduate student preferences. First, the Pythagorean fuzzy preference matrix and attribute weight vectors of graduate students are built considering conformity psychology. Second, the supervisor preference matrix is constructed based on graduate students’ preferences using TODIM. Then, attribute weight vectors of supervisors are determined. On this basis, satisfaction matrices of GSS are set up using TOPSIS^13^ (technique for order preference by similarity to the ideal solution) and grey correlation degrees^14^. Furthermore, a many-to-one GSSM model that considers stability-based fairness is constructed by introducing the matching matrix and the stable matching matrix. A one-to-one GSSM model is constructed by introducing virtual supervisor subjects. Finally, the optimal GSSM scheme is obtained by solving the one-to-one matching model.

The rest of this paper is structured as follows: “Literature review” Section provides an introduction of bilateral matching decision-making (BMDM), mentorship system, Pythagorean fuzzy theory and motivations and contributions of this paper. “Preliminaries” Section outlines relevant theories of LTS, PFN and bilateral matching. “Description of graduate students and supervisors matching problem considering stability-based fairness” Section provided the many-to-one matching problem between GSS considering conformity psychology and graduated students’ preferences. “Matching decision-making for graduate students and supervisors considering stability-based fairness” Section presents a many-to-one matching decision-making method for GSS considering stability-based fairness. “Example analysis” Section demonstrates effectiveness and advantages of the proposed method by an example analysis. “Conclusions” Section concludes this paper.

## Literature review

### Bilateral matching decision-making (BMDM)

The problem of BMDM is prevalent in various aspects of human social life. Early research on BMDM was closely linked to management science, with applications such as marriage matching^15^, university admissions^16^ and job matching^17^. As research has advanced, scholars have broadened theoretical approaches to BMDM. For example, Zhu et al.^18^ proposed a Best–Worst Method (BWM) for rural homesteads exit pattern analysis and the welfare needs of farmers; Xiao^19^ introduced a virtual enterprise partner matching method that incorporates attribute preferences and participant psychological behavior; Qiao and Li^20^ developed a bilateral matching approach based on interval triangular fuzzy sets to match investment funds with financing enterprises; Jia et al.^21^ proposed a BMDM method that incorporates the Choquet integral in the probabilistic linguistic environment; Li et al.^22^ ntroduced a BMDM method for second-hand housing transactions based on an improved decision-making and trial evaluation laboratory within the probabilistic linguistic cloud; Zhang et al.^23^ constructed a BMDM structure model to evaluate the impact of government venture capital; Zhao et al.^24^ developed a person-position matching decision-making method based on probabilistic hesitant fuzzy numbers; Qiao et al.^25^ proposed an interval intuitionistic fuzzy matching decision-making method that maximizes the satisfaction of individual need.

In summary, the existing research had identified numerous practical applications for BMDM. However, the stability-based fairness has been paid limited attention to the study of BMDM. Therefore, designing a BMDM method that considers stability-based fairness is necessary.

### Mentorship system

Existing research on the matching of GSS primarily addresses the mentorship system. The study on mentorship mainly focuses on the field of medical education. For example, Gaeta et al.^26^ reviewed the impact of the current mentorship system on surgical resident burnout and well-being; Diaz-Castrillon et al.^27^ studied the views of medical students to the mentorship system in cardiothoracic surgery; Rajah et al.^28^ evaluated the impact of formal guidance on trainees and the value of mentorship system in promoting employee diversity; Rao et al.^29^ studied the knowledge-transfer mechanisms underlying mentorship-based abuse in hospitality settings; Qureshy et al.^30^ studied the impact of external guidance programs on the career of hematology students; Zhang et al.^31^ studied the impact of academic capital of supervisors on the creativity and perceived mentor support of business graduate students in the context of China’s mentorship system. Additionally, some scholars have started to apply fuzzy decision to the field of university management. For example, Nasution and Nusa^32^ studied the decision making method of Organisasi Intra Sekolah chairperson selection using simple multi attribute rating technique exploiting rank approach and rank order centroid weighting; Maniyan et al.^33^ developed a decision support system that leverages a hybrid data mining model to analyze educational data and unveil concealed patterns and rules; Irvanizam^34^ studied the application of fuzzy TOPSIS multi-attribute decision making method to determine scholarship recipients.

Current research on GSSM primarily focuses on the mentorship system, with relatively limited attention given to methods for matching graduate students with supervisors. Therefore, this paper explores decision-making methods for matching graduate students with supervisors.

### Pythagorean fuzzy theory

Pythagorean fuzzy set is an extension of intuitionistic fuzzy set^35^, which has stronger ability to handle inherent uncertainty and imprecision in decision-making. Fermatean fuzzy set^36,37^ was the extension of Pythagorean fuzzy set. PFNs mainly are employed multi-attribute group decision-making^38,39^. For example, Alballa et al.^40^ studied a potential global supplier optimal selection group decision-making method based on 2-tuple linguistic Pythagorean fuzzy Dombi aggregation operator; Liao and Peng^41^ studied the application of Pythagorean fuzzy measures in multi-attribute decision-making and medical diagnosis; Meng et al.^42^ studied a multi-attribute group decision-making method based on graph neural networks in a Pythagorean fuzzy environment; Mahapatra et al.^43^ proposed a dynamic group decision-making method for enterprise resource planning selection based on the 2-tuple PFN; Li et al.^44^ proposed a comprehensive linguistic Pythagorean fuzzy decision-making method for risk analysis of offshore wind turbines; Gao et al.^45^ developed an integrated 2-tuple linguistic Pythagorean fuzzy decision-making method for operational mechanism evaluation of a single-pilot; Ye et al.^46^ proposed a Pythagorean fuzzy rough set decision-making method with diversified weights; Seikh and Chatterjee^47^ introduced an innovative approach utilizing confidence-based in interval-valued Fermatean fuzzy environment to assess the challenges and find effective strategies for e-waste management.

Pythagorean fuzzy theory has been extensively used in multi-attribute group decision-making; however, its application in GSSM remains relatively under explored. Therefore, this paper studies the multi-attribute matching problem between GSS in the Pythagorean fuzzy environment.

### Motivations and contributions of this paper

In view of the research on Pythagorean fuzzy theory and BMDM, there are the following deficiencies: (1) The application of Pythagorean fuzzy theory in GSSM is relatively limited. (2) The conversion between LTSs and PFNs is rarely studied. (3) In BMDM, the application of conformity psychology still needs further research. (4) The GSSM model considering stability-based fairness has not been established.

Therefore, motivations of this paper are as follows: (1) Applying Pythagorean fuzzy theory to BMDM for graduate students and supervisors can enrich related research. (2) In the process of BMDM, it needs to provide a conversion that covers uncertainty for easily obtained LTS preferences. Thus, it is necessary to propose a method for converting LTSs into PFNs. (3) The psychological behavior of graduate students has a significant impact on bilateral satisfaction levels. Therefore, the conformity psychology and preferences of graduate students deserve more attention. (4) A GSSM model considering stability-based fairness can reveal the uncertainty and ambiguity of BMDM in a more reasonable and detailed way, thereby obtaining a stable and highly fair matching scheme. Therefore, it is necessary to establish a many-to-one BMDM model that considers stability-based fairness.

Based on the above discussion, this paper proposes a many-to-one GSSM decision-making method that considers stability-based fairness. Main contributions of this paper are reflected in theory and practice. Among them, theoretical contributions are reflected in the following aspects:

1. Pythagorean fuzzy theory is applied to GSSM decision-making.
2. An improved score of PFNs is proposed.
3. A method for converting LTSs into PFNs is introduced.
4. Methods for using conformity psychology to adjust initial preferences of graduate students and using graduate students’ preferences to adjust initial preferences of supervisors are proposed.
5. Satisfactions of GSS that combine TOPSIS with grey correlation degrees are calculated.
6. A many-to-one GSSM model considering stability-based fairness is established.

Actual contributions are reflected in following aspects:

1. A BMDM method considering conformity psychology and graduate students’ preferences is proposed to address GSSM problem, which can help improve the effectiveness of GSSM decision-making.
2. A more reasonable model for GSSM is designed that reflect the stability and improve the fairness of matching process, which can help reduce conflicts between GSS and enhances mutual satisfactions.

## Preliminaries

### Linguistic term set and Pythagorean fuzzy number theory

#### Linguistic term set

##### Definition 1^48^

 Let $S = \left\{ {s_{\alpha } |\alpha = 0,1,2, \ldots ,f} \right\}$ be a LTS, where $f$ is an even number, $s_{k}$ represents the $k$-th linguistic term, with $s_{0}$ and $s_{f}$ respectively denoting the lower and upper bounds of $S$. The LTS $S$ must satisfy the following properties: (1) If $\alpha_{1} \le \alpha_{2}$, then $s_{{\alpha_{1} }} \le s_{{\alpha_{2} }}$; (2) There exists an inverse operator ${\text{rec}}(s_{{\alpha_{1} }} ) = s_{{\alpha_{2} }}$ such that $\alpha_{1} { + }\alpha_{2} = g$; (3) If $s_{{\alpha_{1} }} \le s_{{\alpha_{2} }}$, then $\max \left\{ {s_{{\alpha_{1} }} ,s_{{\alpha_{2} }} } \right\} = s_{{\alpha_{2} }}$, $\min \left\{ {s_{{\alpha_{1} }} ,s_{{\alpha_{2} }} } \right\} = s_{{\alpha_{1} }}$.

#### Pythagorean fuzzy number

##### Definition 2^49^

Let $X$ be the domain of discourse and $\vartheta = \left\{ {x,u(x),v(x)|x \in X} \right\}$ be the PFS on $X$, where $u_{\vartheta } (x),v_{\vartheta } (x):X \to \left[ {0,1} \right]$ are the fuzzy sets on $X$, $u_{\vartheta } (x),v_{\vartheta } (x)$ are respectively the membership degree and non-membership degree of $x$ belonging to $\vartheta$, satisfying $u_{\vartheta } (x),v_{\vartheta } (x) \in \left[ {0,1} \right]$, $u_{\vartheta }^{2} (x){ + }v_{\vartheta }^{2} (x) \le 1$, $\forall x \in X$. Let $\pi_{\vartheta } (x) = \sqrt {1 - u_{\vartheta }^{2} (x) - v_{\vartheta }^{2} (x)}$ be the hesitation degree of $x$ belonging to $\vartheta$. If $\forall x \in X$, $\pi_{\vartheta } (x) = 0$, then $\vartheta$ is degenerated into a traditional fuzzy set.

For convenience, an element $\varphi = \left( {u_{\varphi } ,v_{\varphi } } \right)$ of PFS $\vartheta$ is referred to as a PFN.

#### Score of Pythagorean fuzzy number

##### Definition 3^50^

Let $\varphi = \left( {u_{\varphi } ,v_{\varphi } } \right)$ be the PFN, then the score and accuracy of $\varphi$ are respectively defined as:

1 $$S\left( \varphi \right) = u^{2} - v^{2}$$

2 $$H\left( \varphi \right) = u^{2} + v^{2}$$

Let $\varphi_{1} = \left( {u_{{\varphi_{1} }} ,v_{{\varphi_{1} }} } \right)$ and $\varphi_{2} = \left( {u_{{\varphi_{2} }} ,v_{{\varphi_{2} }} } \right)$ be two PFNs, $S\left( {\varphi_{1} } \right)$ and $S\left( {\varphi_{2} } \right)$ represent scores of $\varphi_{1}$ and $\varphi_{2}$ respectively. And $H\left( {\varphi_{1} } \right)$ and $H\left( {\varphi_{2} } \right)$ represent accuracies of $\varphi_{1}$ and $\varphi_{2}$ respectively. Then the comparison rule is expressed as follow:

Rule (i): If $S\left( {\varphi_{1} } \right) \ge S\left( {\varphi_{2} } \right)$, then $\varphi_{1} \ge \varphi_{2}$;

Rule (ii): If $S\left( {\varphi_{1} } \right) = S\left( {\varphi_{2} } \right)$, then when $H\left( {\varphi_{1} } \right) \ge H\left( {\varphi_{2} } \right)$, there is $\varphi_{1} > \varphi_{2}$; and when $H\left( {\varphi_{1} } \right) = H\left( {\varphi_{2} } \right)$, there is $\varphi_{1} = \varphi_{2}$.

##### Example 1

 Let $\varphi_{1} = \left( {0.0387,0.0224} \right)$ and $\varphi_{2} = \left( {0.7070,0.7070} \right)$. Scores $S\left( {\varphi_{1} } \right) = 0.0010 > S\left( {\varphi_{2} } \right) = 0$ and accuracy $H\left( {\varphi_{1} } \right) = 0.0020 < H\left( {\varphi_{2} } \right) = 0.9996$ of them are calculated using Eqs. (1) and (2). Thus $\varphi_{1} > \varphi_{2}$ is obtained according to Rule (i). However, $\left| {S\left( {\varphi_{1} } \right) - S\left( {\varphi_{2} } \right)} \right| = 0.001$, $S\left( {\varphi_{1} } \right)$ and $S\left( {\varphi_{2} } \right)$ are relatively close. ${{H\left( {\varphi_{2} } \right)} \mathord{\left/ {\vphantom {{H\left( {\varphi_{2} } \right)} {H\left( {\varphi_{1} } \right)}}} \right. \kern-0pt} {H\left( {\varphi_{1} } \right)}} \approx 500$. $H\left( {\varphi_{1} } \right)$ is far less than $H\left( {\varphi_{2} } \right)$. Therefore, intuitively speaking, $\varphi_{1} < \varphi_{2}$, which contradicts Rule (i). The primary reason is that the impact of hesitation has not been considered.

In summary, this paper proposes a new score that has strong distinguishing ability.

##### Definition 4

Let $\varphi = \left( {u_{\varphi } ,v_{\varphi } } \right)$ be a PFN. An improved score of $\varphi$ is defined as:

3 $$S^{\varepsilon } \left( \varphi \right) = \left( {\frac{{u^{2} - v^{2} }}{{1{ + }\pi^{2} }}} \right)^{\varepsilon }$$

where $\varepsilon$ is the risk preference coefficient. If $\varepsilon < 1$, it indicates the risk-averse behavior; if $\varepsilon = 1$, it indicates the risk-neutral behavior; if $\varepsilon > 1$, it indicates the risk-seeking behavior.

Let two PFNs $\varphi_{1} = \left( {u_{{\varphi_{1} }} ,v_{{\varphi_{1} }} } \right)$ and $\varphi_{2} = \left( {u_{{\varphi_{2} }} ,v_{{\varphi_{2} }} } \right)$, the scores of $\varphi_{1}$ and $\varphi_{2}$ be $S^{\varepsilon } \left( {\varphi_{1} } \right)$ and $S^{\varepsilon } \left( {\varphi_{2} } \right)$ respectively. Then a new comparison rule is expressed as follow:

Rule (iii): If $S^{\varepsilon } \left( {\varphi_{1} } \right) \ge S^{\varepsilon } \left( {\varphi_{2} } \right)$, then $\varphi_{1} \ge \varphi_{2}$.

##### Example 2

 For PFNs $\varphi_{1} = \left( {0.7,0.6} \right)$ and $\varphi_{2} = \left( {0.8,0.5} \right)$ in Example 1, new scores $S^{1} \left( {\varphi_{1} } \right) = 0.05$ and $S^{1} \left( {\varphi_{2} } \right) = 0.21$ can be calculated by using Eq. (3). Then, according to Rule (iii), $\varphi_{1} < \varphi_{2}$ is obtained because of $S^{1} \left( {\varphi_{1} } \right) < S^{1} \left( {\varphi_{2} } \right)$.

#### Pythagorean fuzzy distance

##### Definition 5

 Let $\varphi = \left( {u_{\varphi } ,v_{\varphi } } \right)$ and $\varphi_{2} = \left( {u_{{\varphi_{2} }} ,v_{{\varphi_{2} }} } \right)$ be two PFNs, then the $\varpi$-norm index distance between $\varphi_{1}$ and $\varphi_{2}$ is defined as:

4 $$d^{\varpi } \left( {\varphi_{1} ,\varphi_{2} } \right) = \frac{1}{2}\left( {\left| {u_{{\varphi_{1} }}^{2} - u_{{\varphi_{2} }}^{2} } \right|^{\varpi } + \left| {\nu_{{\varphi_{1} }}^{2} - \nu_{{\varphi_{2} }}^{2} } \right|^{\varpi } + \left| {\pi_{{\varphi_{1} }}^{2} - \pi_{{\varphi_{2} }}^{2} } \right|^{\varpi } } \right)^{{{1 \mathord{\left/ {\vphantom {1 \varpi }} \right. \kern-0pt} \varpi }}}$$

where $\varpi$ as the norm index of distance. When $\varpi = 1$, $d^{1} \left( {\varphi_{1} ,\varphi_{2} } \right)$ is the Manhattan distance. When $\varpi = 2$, $d^{2} \left( {\varphi_{1} ,\varphi_{2} } \right)$ is the Euclidean distance. When $\varpi \to \infty$, $d^{\infty } \left( {\varphi_{1} ,\varphi_{2} } \right)$ is the Chebyshev distance.

#### Method for converting linguistic term sets into Pythagorean fuzzy numbers

##### Definition 6

Let $S = \left\{ {s_{\alpha } |\alpha = 0,1,2,...,f} \right\}$ be a LTS, then $s_{k}$ can be converted into the following PFN $\left( {u_{k} ,v_{k} } \right)$:

5 $$\left( {u_{k} ,v_{k} } \right) = \left( {\sqrt {\left( \frac{k}{f} \right)^{2} - g\pi^{2} } ,\sqrt {\left( {\frac{{\sqrt {\left( {2kf - k^{2} } \right)} }}{f}} \right)^{2} - h\pi^{2} } } \right)$$

where $\pi^{2}$ represents the level of hesitation, $0 \le \pi^{2} \le 1$; $g$ and $h$ represent adjustment coefficients, which are used to adjust hesitations between membership and non-membership, satisfying $g{ + }h = 1$.

##### Example 3

 Assuming that $S = \left\{ {s_{\alpha } |\alpha = 0,1,2, \ldots ,6} \right\}$, $s_{5}$ is transformed into PFN $\left( {u_{5} ,v_{5} } \right) = (0.81,0.49)$ by using Eq. (5). Here $\pi^{2} = 0.1$, $g = {1 \mathord{\left/ {\vphantom {1 3}} \right. \kern-0pt} 3}$, $h = {2 \mathord{\left/ {\vphantom {2 3}} \right. \kern-0pt} 3}$.

### Bilateral matching theory

#### Bilateral matching

Let one set of subjects be $\vartheta = \left\{ {\vartheta_{1} ,\vartheta_{2} , \ldots ,\vartheta_{m} } \right\}$, where $\vartheta_{i}$ represents the $i$-th subject, $i \in M = \left\{ {1,2, \ldots ,m} \right\}$; the other set of subjects be $\chi = \left\{ {\chi_{1} ,\chi_{2} , \ldots ,\chi_{n} } \right\}$, where $\chi_{j}$ represents the $j$-th subject, $j \in N = \left\{ {1,2, \ldots ,n} \right\}$, $m \le n$.

##### Definition 7^22^

Let $\varphi$ be a one-to-one mapping: $\vartheta \cup \chi \to \chi \cup \vartheta$. It satisfies the following conditions: (a) $\varphi (\vartheta_{i} ) \in \chi$; (b) $\varphi (\chi_{j} ) \in \vartheta \cup \left\{ {\chi_{j} } \right\}$; (c) $\varphi (\vartheta_{i} ) = \chi_{j}$ if and only if $\varphi (\chi_{j} ) = \vartheta_{i}$; (d) if $\tilde{\varphi }(\chi_{j} ) = \vartheta_{i}$, then $\tilde{\varphi }(\chi_{f} ) \ne \vartheta_{i} ,\forall f \in N,j \ne f$; (e) if $\tilde{\varphi }(\vartheta_{i} ) = \chi_{j}$, then $\tilde{\varphi }(\vartheta_{l} ) \ne \chi_{j} ,\forall l \in M,l \ne i$. Then $\varphi$ is called one-to-one bilateral matching. Here, $\varphi (\vartheta_{i} ) = \chi_{j}$ represents that $\vartheta_{i}$ and $\chi_{j}$ are matched, and $\varphi (\chi_{j} ) = \chi_{j}$ represents $\chi_{j}$ unmatched or single.

##### Definition 8^51^

Let $\tilde{\varphi }$ be a many-to-one mapping: $\vartheta \cup \chi \to \chi \cup \vartheta$. It satisfies the following conditions: (a) $\tilde{\varphi }(\vartheta_{i} ) \in \vartheta_{i} \cup \chi$; (b) $\tilde{\varphi }(\chi_{j} ) \in \vartheta \cup \chi_{j}$; (c) if $\tilde{\varphi }(\chi_{j} ) = \left\{ {\vartheta_{{i_{1} }} ,\vartheta_{{i_{2} }} , \ldots ,\vartheta_{{i_{p} }} } \right\}$, where $i_{1} ,i_{2} , \ldots ,i_{p} \in M$, then $\tilde{\varphi }(\vartheta_{{i_{1} }} ) = \tilde{\varphi }(\vartheta_{{i_{2} }} ) = \cdots = \tilde{\varphi }(\vartheta_{{i_{p} }} ) = \chi_{j}$; d) $\tilde{\varphi }(\vartheta_{i} ) = \chi_{j}$ if and only if $\tilde{\varphi }(\chi_{j} ) = \vartheta_{i}$; (e) $\tilde{\varphi }(\chi_{j} ) \cap \tilde{\varphi }(\chi_{f} ) = \emptyset ,\forall f \in N,j \ne f$. Then $\varphi$ is called a many-to-one bilateral matching.

#### One-on-one stable matching

##### Definition 9^52^

Let $\varphi$ be a one-to-one bilateral matching, $a_{ij}$ be the satisfaction of subject $\vartheta_{i}$ for $\chi_{j}$, and $b_{ij}$ be the satisfaction of subject $\chi_{j}$ for $\vartheta_{i}$. If the matching pair $(\vartheta_{i} ,\chi_{j} )$ satisfies one of the following conditions:

- $$\exists \vartheta_{l} \in \vartheta ,\chi_{s} \in \chi ,\varphi (\vartheta_{i} ) = \chi_{s} ,\varphi (\chi_{j} ) = \vartheta_{l} ;$$ and $$a_{ij} \ge a_{is}$$,$$b_{ij} \ge b_{lj}$$;
- $$\exists \chi_{s} \in \chi ,\varphi (\vartheta_{i} ) = \chi_{s} ,\varphi (\chi_{j} ) = \chi_{j} ;$$ and $$a_{ij} \ge a_{is}$$;
- $$\exists \vartheta_{l} \in \vartheta ,\varphi (\vartheta_{i} ) = \vartheta_{i} ,\varphi (\chi_{j} ) = \vartheta_{l} ;$$ and $$b_{ij} \ge b_{lj}$$.

Here $(\vartheta_{i} ,\chi_{j} )$ is called an obstacle pair. If there are no obstacle pairs in $\varphi$, then $\varphi$ is called a one-to-one stable matching.

#### Stability-based fairness

##### Definition 10

 Let $\Upsilon = \left\{ {\left( {\vartheta_{1} ,\varphi (\vartheta_{1} )} \right),\left( {\vartheta_{2} ,\varphi (\vartheta_{2} )} \right), \ldots ,\left( {\vartheta_{m} ,\varphi (\vartheta_{m} )} \right)} \right\}$ be a matching scheme, $a_{i\varphi (i)}$ be the satisfaction of subject $\vartheta_{i}$ for $\chi_{\varphi (i)}$, and $b_{i\varphi (i)}$ be the satisfaction of subject $\chi_{\varphi (i)}$ for $\vartheta_{i}$. If matching pairs $\left( {\vartheta_{i} ,\varphi (\vartheta_{i} )} \right)(i = 1,2,...,m)$ in $\Upsilon$ are not obstacle pairs, then $\Upsilon$ is called a stable matching scheme. Then $\upsilon_{\Upsilon }$ is referred to as a stability-based fairness of $\Upsilon$ and expressed as:

6 $$\upsilon_{\Upsilon } = \sum\limits_{\varphi (i)} {\sum\limits_{i = 1}^{m} {\frac{{\mathop {\max }\limits_{i \in M} (a_{i\varphi (i)} - b_{i\varphi (i)} )^{2} - (a_{i\varphi (i)} - b_{i\varphi (i)} )^{2} }}{{\mathop {\max }\limits_{i \in M} (a_{i\varphi (i)} - b_{i\varphi (i)} )^{2} - \mathop {\min }\limits_{i \in M} (a_{i\varphi (i)} - b_{i\varphi (i)} )^{2} }}} }$$

Obviously, the greater the fairness $\upsilon_{\Upsilon }$, the better the stable matching scheme $\Upsilon$.

## Description of graduate students and supervisors matching problem considering stability-based fairness

Regarding the GSSM problem in the university, assume that the set of graduate students is $\vartheta = \left\{ {\vartheta_{1} ,\vartheta_{2} , \ldots ,\vartheta_{m} } \right\}$, where graduate student $\vartheta_{i}$ can only be matched with one supervisor; the set of supervisors is $\chi = \left\{ {\chi_{1} ,\chi_{2} , \ldots ,\chi_{n} } \right\}$, and the number of graduate students that supervisor $\chi_{j}$ can be matched with is $c_{j}$, $m \ge n$. The attribute set for graduate students evaluating supervisors is $\gamma^{\vartheta } = \left\{ {\gamma_{1}^{\vartheta } ,\gamma_{2}^{\vartheta } , \ldots ,\gamma_{\kappa }^{\vartheta } } \right\}$, where $\gamma_{t}^{\vartheta }$ represents the $t$-th attribute, $t = 1,2, \ldots ,\kappa$; the set of attributes evaluated by supervisors for graduate students is $\gamma^{\chi } = \left\{ {\gamma_{1}^{\chi } ,\gamma_{2}^{\chi } , \ldots ,\gamma_{p}^{\chi } } \right\}$, where $\gamma_{l}^{\chi }$ represents the $l$-th attribute, $l = 1,2,...,p$. Assume that the weight vector of attribute set $\gamma^{\vartheta }$ for graduate student $\vartheta_{i}$ is $W^{{\vartheta_{i} }} = \left( {w_{1}^{{\vartheta_{i} }} ,w_{2}^{{\vartheta_{i} }} , \ldots ,w_{\kappa }^{{\vartheta_{i} }} } \right)$, satisfying $0 \le w_{t}^{{\vartheta_{i} }} \le 1$, $\sum\limits_{t = 1}^{\kappa } {w_{t}^{{\vartheta_{i} }} } = 1$, $i \in M$; the weight vector of attribute set $\gamma^{\chi }$ for supervisor $\chi_{j}$ is $W^{{\chi_{j} }} = \left( {w_{1}^{{\chi_{j} }} ,w_{2}^{{\chi_{j} }} , \ldots ,w_{p}^{{\chi_{j} }} } \right)$, which satisfies $0 \le w_{l}^{{\chi_{j} }} \le 1$, $\sum\limits_{l = 1}^{p} {w_{l}^{{\chi_{j} }} } = 1$. Assume that the linguistic term matrix (LTM) given by graduate students to supervisors under attribute set $\gamma^{\vartheta }$ is $S^{\vartheta } = \left[ {s_{itj}^{\vartheta } } \right]_{m \times \kappa \times n}$, where $s_{ijt}^{\vartheta } \in S = \left\{ {s_{\alpha } |\alpha = 0,1,2, \ldots ,f} \right\}$; the LTM given by supervisors to graduate student under attribute set $\gamma^{\chi }$ is $S^{\chi } = \left[ {s_{jli}^{\chi } } \right]_{n \times p \times m}$, where $s_{jli}^{\chi } \in S = \left\{ {s_{\alpha } |\alpha = 0,1,2, \ldots ,f} \right\}$.

*Note 1* Based on attribute comparison information, the conformity coefficient matrix, the preference coefficient matrix and attribute weight vectors $W^{{\vartheta_{i} }} (i \in M)$ and $W^{{\chi_{j} }} (j \in N)$ of GSS can be determined using the BWM method, as detailed in “Calculation of attribute weight vectors of supervisors” Section.

Based on the above analysis, this paper aims to solve the following problems in sequence: (1) How to calculate attribute weight vectors $W^{{\vartheta_{i} }} (i \in M)$ and $W^{{\chi_{j} }} (j \in N)$ according to the attribute comparison information, conformity coefficient matrix $V = \left[ {\wp_{ik} } \right]_{m \times m}$, and preference coefficient matrix $U = \left[ {\mu_{ij}^{p} } \right]_{m \times n}$; (2) how to calculate satisfactions of graduate students considering conformity psychology and satisfactions of supervisors considering graduate students’ preferences according to linguistic term matrices $S^{\vartheta } = \left[ {s_{itj}^{\vartheta } } \right]_{m \times \kappa \times n}$ and $S^{\chi } = \left[ {s_{jli}^{\chi } } \right]_{n \times p \times m}$, the conformity coefficient matrix $V = \left[ {\wp_{ik} } \right]_{m \times m}$, preference coefficient matrix $U = \left[ {\mu_{ij}^{p} } \right]_{m \times n}$ and attribute weight vectors $W^{{\vartheta_{i} }} (i \in M)$ and $W^{{\chi_{j} }} (j \in N)$; (3) how to set up a many-to-one matching model considering stability-based fairness, and solve the model to obtain the optimal matching scheme $\Upsilon$ between GSS according to satisfaction matrices of GSS $A = \left[ {a_{ij} } \right]_{m \times n}$ and $B = \left[ {b_{ij} } \right]_{m \times n}$. The main acronyms for this paper are displayed in Table 1

**Table 1:** List of abbreviations.

Acronym	Meaning
GSSM	Graduate students and supervisors matching
GSS	Graduate students and supervisors
LTM	linguistic term matrix
LTS	Linguistic term set
PFM	Pythagorean fuzzy matrix
PFN	Pythagorean fuzzy number
BWM	Best–worst method
TODIM	A Portuguese acronym for interactive and multicriteria decision making
TOPSIS	Technique for order preference by similarity to the ideal solution
BMDM	Bilateral matching decision-making
BPF	Benefit Pythagorean fuzzy

## Matching decision-making for graduate students and supervisors considering stability-based fairness

**Fig. 1:**
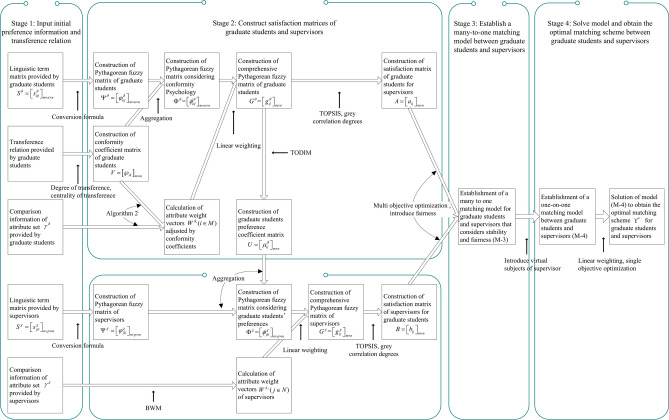
Solution idea for many-to-one GSSM decision-making considering stability-based fairness.

The decision-making steps for matching GSS are displayed as follow: GSS $\Psi^{\vartheta } = \left[ {\varphi_{itj}^{\vartheta } } \right]_{m \times \kappa \times n}$ of graduate students is established according to the LTM $S^{\vartheta } = \left[ {s_{itj}^{\vartheta } } \right]_{m \times \kappa \times n}$ provided by graduate students; the conformity coefficient matrix $V = \left[ {\wp_{ik} } \right]_{m \times m}$ of graduate students is built according to the transference relation of graduate students. On this basis, the Pythagorean fuzzy matrix (PFM) considering conformity psychology $\Phi^{\vartheta } = \left[ {\phi_{itj}^{\vartheta } } \right]_{m \times \kappa \times n}$ is constructed; based on attribute comparison information provided by graduate students and the conformity coefficient matrix $V = \left[ {\wp_{ik} } \right]_{m \times m}$, attribute weight vectors $W^{{\vartheta_{i} }} (i \in M)$ of graduate students are determined by using Algorithm 2; the comprehensive PFM $G^{\vartheta } = \left[ {g_{ij}^{\vartheta } } \right]_{m \times n}$ of graduate students is calculated by aggregating matrix $\Phi^{\vartheta } = \left[ {\phi_{itj}^{\vartheta } } \right]_{m \times \kappa \times n}$ and attribute weight vectors $W^{{\vartheta_{i} }} (i \in M)$. Then, the satisfaction matrix $A = \left[ {a_{ij} } \right]_{m \times n}$ of graduate students is set up using TOPSIS and grey correlation degrees. On the other hand, the PFM $\Psi^{\chi } = \left[ {\varphi_{jli}^{\chi } } \right]_{n \times p \times m}$ of supervisors is established based on the LTM $S^{\chi } = \left[ {s_{ilj}^{\chi } } \right]_{m \times p \times n}$; the preference coefficient matrix $U = \left[ {\mu_{ij}^{p} } \right]_{m \times n}$ of graduate students is built by using TODIM according to the comprehensive PFM $G^{\vartheta } = \left[ {g_{ij}^{\vartheta } } \right]_{m \times n}$; the PFM $\Phi^{\chi } = \left[ {\phi_{jli}^{\chi } } \right]_{n \times p \times m}$ considering graduate students’ preferences is constructed based on matrices $\Psi^{\chi } = \left[ {\varphi_{jli}^{\chi } } \right]_{n \times p \times m}$ and $U = \left[ {\mu_{ij}^{p} } \right]_{m \times n}$; Based on attribute comparison information provided by supervisors, attribute weight vectors $W^{{\chi_{j} }} (j \in N)$ of supervisors are determined using Algorithm 5; the comprehensive PFM $G^{\chi } = \left[ {g_{ij}^{\chi } } \right]_{m \times n}$ of supervisors is constructed by aggregating matrix $\Phi^{\chi } = \left[ {\phi_{jli}^{\chi } } \right]_{n \times p \times m}$ and attribute weight vectors $W^{{\chi_{j} }} (j \in N)$. On this basis, the satisfaction matrix $B = \left[ {b_{ij} } \right]_{m \times n}$ of supervisors is established using TOPSIS and grey correlation degrees. Furthermore, a many-to-one GSSM model considering stability-based fairness is set up according to satisfaction matrices $A = \left[ {a_{ij} } \right]_{m \times n}$ and $B = \left[ {b_{ij} } \right]_{m \times n}$ of bilateral subjects. Then the many-to-one bilateral matching model is transformed into a one-to-one matching model by introducing virtual supervisor subjects. Finally, the model is solved to obtain the optimal matching scheme between GSS. The detailed process is shown in Fig. 1.

### Construction of the satisfaction matrix of graduate students for supervisors

#### Construction of Pythagorean fuzzy matrix considering conformity psychology

Graduate student communities exhibit pronounced conformity behaviors, where both conformity and transference relations capture individuals’ attention to others’ decisions. Thus, this paper employs directed transference graph to formalize transference relation and calculate conformity coefficients of graduate students. The construction process of the PFM $\Phi^{\vartheta } = \left[ {\phi_{itj}^{\vartheta } } \right]_{m \times \kappa \times n}$ incorporating conformity psychology is displayed as follows:

First, based on the LTM $S^{\vartheta } = \left[ {s_{itj}^{\vartheta } } \right]_{m \times \kappa \times n}$ provided by graduate students, the PFM $\Psi^{\vartheta } = \left[ {\varphi_{itj}^{\vartheta } } \right]_{m \times \kappa \times n}$ of graduate students is established using Eq. (5).

Second, the directed transference graph $R = \left[ {\Im_{ik} ,r(i,k)} \right]_{m \times m}$ is used to represent transference relation among the community of graduate students. Where each node $\vartheta_{i}$ represents graduate student $\vartheta_{i}$; directed arc $r(i,k)$ represents the direction of transference relation of node $\vartheta_{i}$ to $\vartheta_{k}$; $\Im_{ii}$ represents the degree of node $\vartheta_{i}$ adheres to its own decision results; $\Im_{ik}$ represents the degree of transference of node $\vartheta_{i}$ towards $\vartheta_{k}$,

satisfying $\Im_{ii} { + }\sum\limits_{i \ne k} {\Im_{ik} } = 1$. In addition, the centrality $\Im C_{i}$ of node $\vartheta_{i}$ in the directed transference graph represents the influence of graduate students on the decisions of others and.

is expressed as:

7 $$\Im C_{i} = \Im_{ii} + \sum\limits_{i \ne k} {\Im_{ki} }$$

Obviously, the larger the centrality $\Im C_{i}$ is, the more important node $\vartheta_{i}$ is in the directed transference graph; that is, the greater the influence of graduate student $\vartheta_{i}$ on the decisions of others.

Third, the conformity relation $\Re_{ik}$ between node $\vartheta_{i}$ and $\vartheta_{k}$ based on the degree of transference $\Im_{ik}$ and centrality $\Im C_{i}$ is calculated as:

8 $$\Re_{ik} = e\Im_{ik} + (1 - e)C\Im_{i}$$

where $e$ is the adjustment coefficient of conformity relation $\Re_{ik}$, which is used to regulate the importance of degree of transference and centrality of transference in conformity relation $\Re_{ik}$, satisfying $0 \le e \le 1$.

Fourth, the conformity coefficient $\wp_{ik}$ of node $\vartheta_{i}$ to $\vartheta_{k}$ is calculated by normalizing the conformity relation $\Re_{ik}$ and expressed as:

9 $$\wp_{ik} = \frac{{\Re_{ik} }}{{\sum\limits_{k} {\Re_{ik} } }}$$

Then, the conformity coefficient matrix $V = \left[ {\wp_{ik} } \right]_{m \times m}$ is set up. Based on PFM $\Psi^{\vartheta } = \left[ {\varphi_{itj}^{\vartheta } } \right]_{m \times \kappa \times n}$ and conformity coefficient matrix $V = \left[ {\wp_{ik} } \right]_{m \times m}$, the PFM $\Phi^{\vartheta } = \left[ {\phi_{itj}^{\vartheta } } \right]_{m \times \kappa \times n}$ considering conformity psychology is constructed, where $\phi_{itj}^{\vartheta }$ is calculated as:

10 $$\phi_{itj}^{\vartheta } = \left( {\varphi_{itj}^{\vartheta } } \right)^{\xi } \mathop \Pi \limits_{k} \left( {\varphi_{ktj}^{\vartheta } } \right)^{{\left( {1 - \xi } \right)\wp_{ik} }} = \left\{ {\left( {\left( {u_{itj}^{\vartheta } } \right)^{\zeta } \left( {\mathop \Pi \limits_{{k:u_{ktj}^{\vartheta } \ne 0}} \left( {u_{ktj}^{\vartheta } } \right)^{{\wp_{ik} }} } \right)^{{\left( {1 - \zeta } \right)}} ,\left( {v_{itj}^{\vartheta } } \right)^{\zeta } \left( {\mathop \Pi \limits_{{k:v_{ktj}^{\vartheta } \ne 0}} \left( {v_{ktj}^{\vartheta } } \right)^{{\wp_{ik} }} } \right)^{{\left( {1 - \zeta } \right)}} } \right)} \right.$$

In Eq. (10), $\zeta$ is the decision adjustment coefficient, which used to regulate the proportion of graduate students’ internal decisions and others decisions, satisfying $0 \le \zeta \le 1$.

*Note 2*: In the actual GSSM problem, attributes considered by graduate students in this paper include professional title, scholarly output, personal style and research direction. All these attributes are categorized as benefit attributes. Therefore, the solution process outlined above does not incorporate methods for handling cost attributes. If cost attributes exist in practical scenarios, PFM $\Phi^{\vartheta } = \left[ {\phi_{itj}^{\vartheta } } \right]_{m \times \kappa \times n}$ can be converted into the benefit Pythagorean fuzzy (BPF) matrix $\Phi^{\prime \vartheta } = \left[ {\phi_{itj}^{\prime \vartheta } } \right]_{m \times \kappa \times n}$ using Eq. (11), where $\phi_{itj}^{\prime \vartheta }$ is computed as:

11 $$\phi_{itj}^{\prime \vartheta } = \left\{ \begin{gathered} \phi_{itj}^{\vartheta } ,{\text{ benefit attribute }} \hfill \\ \overline{\phi }_{itj}^{\vartheta } ,{\text{ cost attribute}} \hfill \\ \end{gathered} \right.$$

In Eq. (11), when attribute $\chi_{t}$ is the benefit one, the BPF number corresponding to $\phi_{itj}^{\vartheta }$ is itself. When attribute $\chi_{t}$ is the cost one, the complement^44^ of BPF number corresponding to $\phi_{itj}^{\vartheta }$ is $\overline{\phi }_{itj}^{\vartheta }$.

In summary, an algorithm for establishing a PFM $\Phi^{\vartheta } = \left[ {\phi_{itj}^{\vartheta } } \right]_{m \times \kappa \times n}$ considering conformity psychology is developed and its pseudocode is displayed as follows:

**Algorithm 1:**
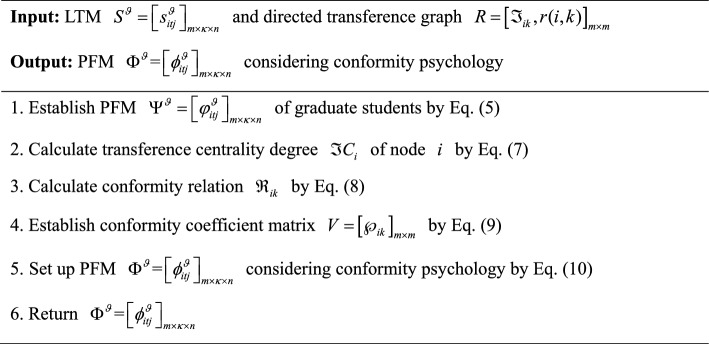
Construction of the PFM $\Phi ^{\vartheta } = \left[ {\phi _{{itj}}^{\vartheta } } \right]_{{m \times \kappa \times n}}$ considering conformity psychology

**Fig. 2:**
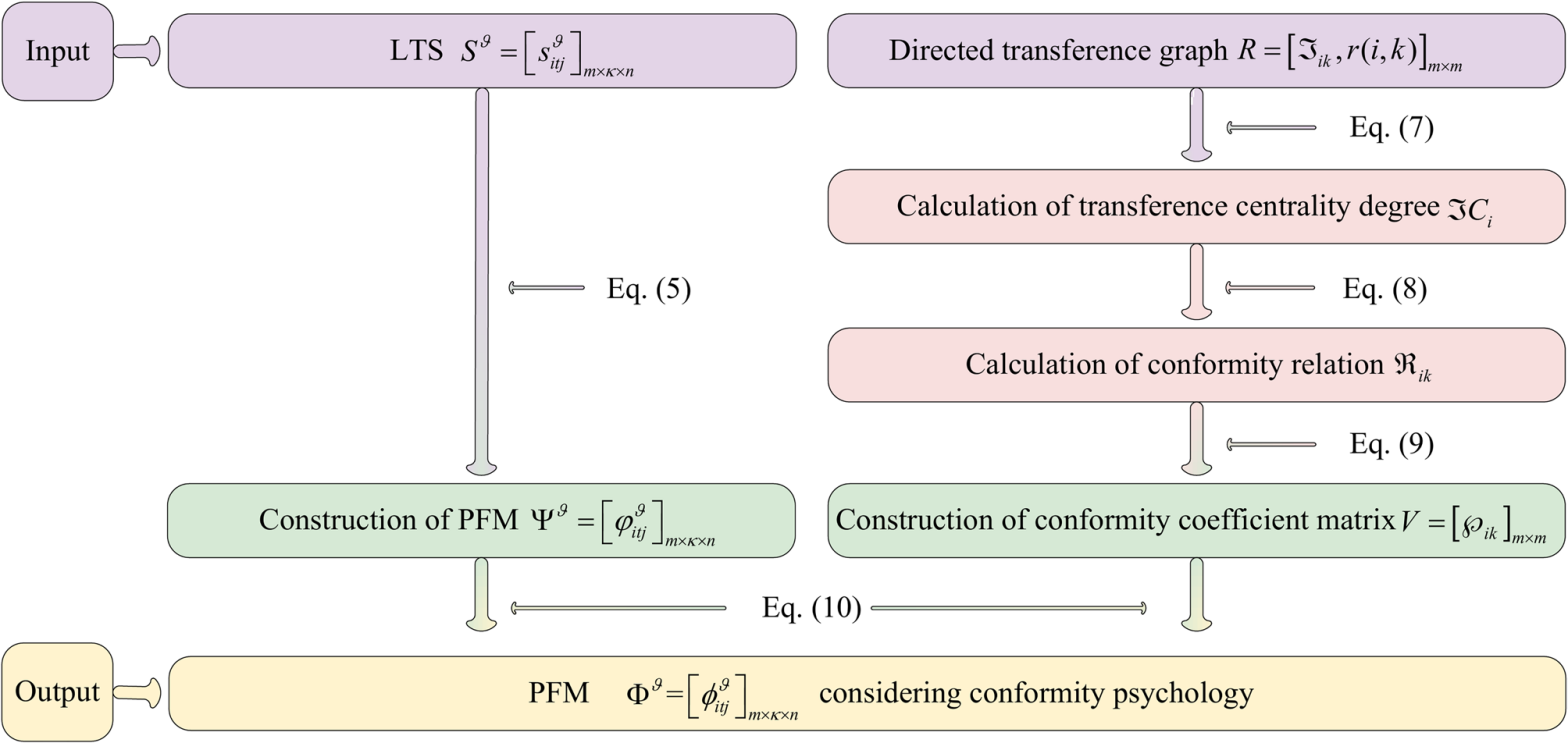
Flowchart of Algorithm 1.

To provide a more intuitive description of Algorithm 1, it is visually presented by Fig. 2.

#### Calculation of attribute weight vectors considering conformity coefficients

BWM attribute weight vectors $\tilde{W}^{{\vartheta_{i} }} = \left( {\tilde{w}_{1}^{{\vartheta_{i} }} ,\tilde{w}_{2}^{{\vartheta_{i} }} , \ldots ,\tilde{w}_{\kappa }^{{\vartheta_{i} }} } \right)\left( {i \in M} \right)$ will be calculated based on attribute comparison information; then attribute weight vectors $W^{{\vartheta_{i} }} = \left( {w_{1}^{{\vartheta_{i} }} ,w_{2}^{{\vartheta_{i} }} , \ldots ,w_{\kappa }^{{\vartheta_{i} }} } \right)\left( {i \in M} \right)$ of graduate students considering conformity coefficients will be computed based on conformity coefficient matrix $V = \left[ {\wp_{ik} } \right]_{m \times m}$ and $\tilde{W}^{{\vartheta_{i} }} = \left( {\tilde{w}_{1}^{{\vartheta_{i} }} ,\tilde{w}_{2}^{{\vartheta_{i} }} , \ldots ,\tilde{w}_{\kappa }^{{\vartheta_{i} }} } \right)\left( {i \in M} \right)$. The solving process is as follows:

First, the optimal attribute $\gamma_{o}^{{\vartheta_{i} }}$ and the worst attribute $\gamma_{w}^{{\vartheta_{i} }}$ from attribute set $\gamma^{\vartheta }$ are selected by graduate student $\vartheta_{i}$; Then, comparison vector $H_{o}^{{\vartheta_{i} }} = \left( {h_{o1}^{{\vartheta_{i} }} ,h_{o1}^{{\vartheta_{i} }} , \ldots ,h_{o\kappa }^{{\vartheta_{i} }} } \right)$ of the optimal attribute $\gamma_{o}^{{\vartheta_{i} }}$ compared to other attributes $\gamma_{l}^{{\vartheta_{i} }} (l = 1,2, \ldots ,\kappa )$ are constructed by graduate student $\vartheta_{i}$ using the 1–9 scale, and comparison vector $H_{w}^{{\vartheta_{i} }} = \left( {h_{1w}^{{\vartheta_{i} }} ,h_{2w}^{{\vartheta_{i} }} , \ldots ,h_{\kappa w}^{{\vartheta_{i} }} } \right)$ of other attributes $\gamma_{l}^{{\vartheta_{i} }} (l = 1,2, \ldots ,\kappa )$ compared to the worst attribute $\gamma_{w}^{{\vartheta_{i} }}$ are constructed by graduate student $\vartheta_{i}$ using the 1–9 scale. The 1–9 scale is represented in Table 2.

**Table 2:** Meanings of the 1–9 scale of comparison vector.

Scale	Meaning
1	Attribute $\gamma_{a}$ is equally important compared to $\gamma_{h}$
3	Attribute $\gamma_{a}$ is slightly more important compared to $\gamma_{h}$
5	Attribute $\gamma_{a}$ is significantly more important compared to $\gamma_{h}$
7	Attributes $\gamma_{a}$ are very important compared to $\gamma_{h}$
9	Attribute $\gamma_{a}$ is extremely important compared to $\gamma_{h}$
2, 4, 6, 8	median of adjacent judgments

Second, the following mathematical programming model (M-1) is constructed based on the information given by graduated student $\vartheta_{i}$:

$$(M - 1)\left\{ \begin{gathered} Min\delta \hfill \\ s.t.\left| {\frac{{\tilde{w}_{o}^{{\vartheta_{i} }} }}{{\tilde{w}_{h}^{{\vartheta_{i} }} }} - h_{oh}^{{\vartheta_{i} }} } \right| \le \iota ,h = 1,2, \ldots ,\kappa ; \hfill \\ \left| {\frac{{\tilde{w}_{h}^{{\vartheta_{i} }} }}{{\tilde{w}_{w}^{{\vartheta_{i} }} }} - h_{hw}^{{\vartheta_{i} }} } \right| \le \iota ,h = 1,2, \ldots ,\kappa ; \hfill \\ \sum\limits_{h = 1}^{\kappa } {\tilde{w}_{h}^{{\vartheta_{i} }} } = 1,\tilde{w}_{h}^{{\vartheta_{i} }} \ge 0,h = 1,2, \ldots ,\kappa . \hfill \\ \end{gathered} \right.$$

Third, model (M-1) is solved by Lingo 11 (URL: https://lindo.com/index.php/ls-downloads/try-lingo) to obtain BWM attribute weight vector $\tilde{W}^{{\vartheta_{i} }} = \left( {\tilde{w}_{1}^{{\vartheta_{i} }} ,\tilde{w}_{2}^{{\vartheta_{i} }} , \ldots ,\tilde{w}_{\kappa }^{{\vartheta_{i} }} } \right)$ for graduate student $\vartheta_{i}$ with respect to the attribute set $\gamma^{\vartheta }$.

Fourth, BWM attribute weight vector $\tilde{W}^{{\vartheta_{i} }} = \left( {\tilde{w}_{1}^{{\vartheta_{i} }} ,\tilde{w}_{2}^{{\vartheta_{i} }} , \ldots ,\tilde{w}_{\kappa }^{{\vartheta_{i} }} } \right)$ and conformity coefficient matrix $V = \left[ {\wp_{ik} } \right]_{m \times m}$ are aggregated to obtain $\overline{w}_{l}^{{\vartheta_{i} }}$, where $\overline{w}_{l}^{{\vartheta_{i} }}$ is presented as:

12 $$\overline{w}_{l}^{{\vartheta_{i} }} = \left( {\tilde{w}_{l}^{{\vartheta_{i} }} } \right)^{\xi } \mathop \Pi \limits_{k} \left( {\tilde{w}_{l}^{{\vartheta_{k} }} } \right)^{{\left( {1 - \xi } \right)\wp_{ik} }}$$

where $\xi$ is the attribute weight adjustment coefficient and used to adjust the proportion of internal evaluation of graduate student $\vartheta_{i}$ and others evaluations, satisfying $0 \le \xi \le 1$.

Finally, $\overline{w}_{l}^{{\vartheta_{i} }}$ is standardized to obtain attribute weight vector $W^{{\vartheta_{i} }} = \left( {w_{1}^{{\vartheta_{i} }} ,w_{2}^{{\vartheta_{i} }} , \ldots ,w_{\kappa }^{{\vartheta_{i} }} } \right)$ considering conformity coefficients, where $w_{l}^{{\vartheta_{i} }}$ is calculated as:

13 $$w_{l}^{{\vartheta_{i} }} = \frac{{\overline{w}_{l}^{{\vartheta_{i} }} }}{{\sum\limits_{l = 1}^{p} {\overline{w}_{l}^{{\vartheta_{i} }} } }}$$

In summary, an algorithm for calculating attribute weight vectors $W^{{\vartheta_{i} }} \left( {i \in M} \right)$ considering conformity coefficients is developed and its pseudocode is displayed as follows:

**Algorithm 2:**
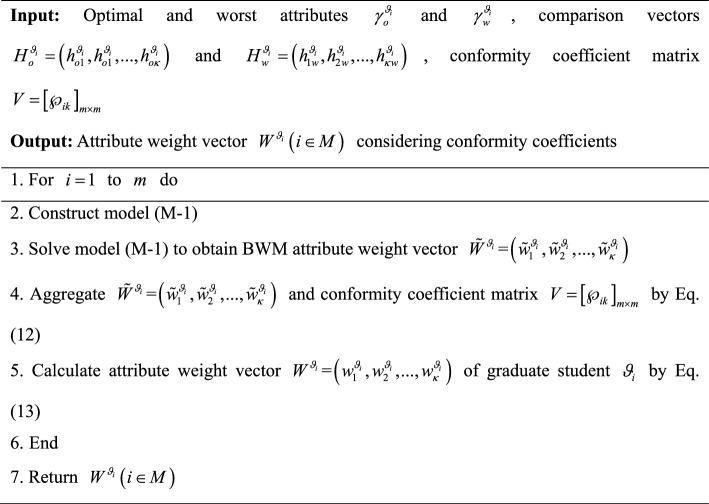
Calculation of attribute weight vectors $W^{{\vartheta _{i} }} \left( {i \in M} \right)$ considering conformity coefficients

**Fig. 3:**
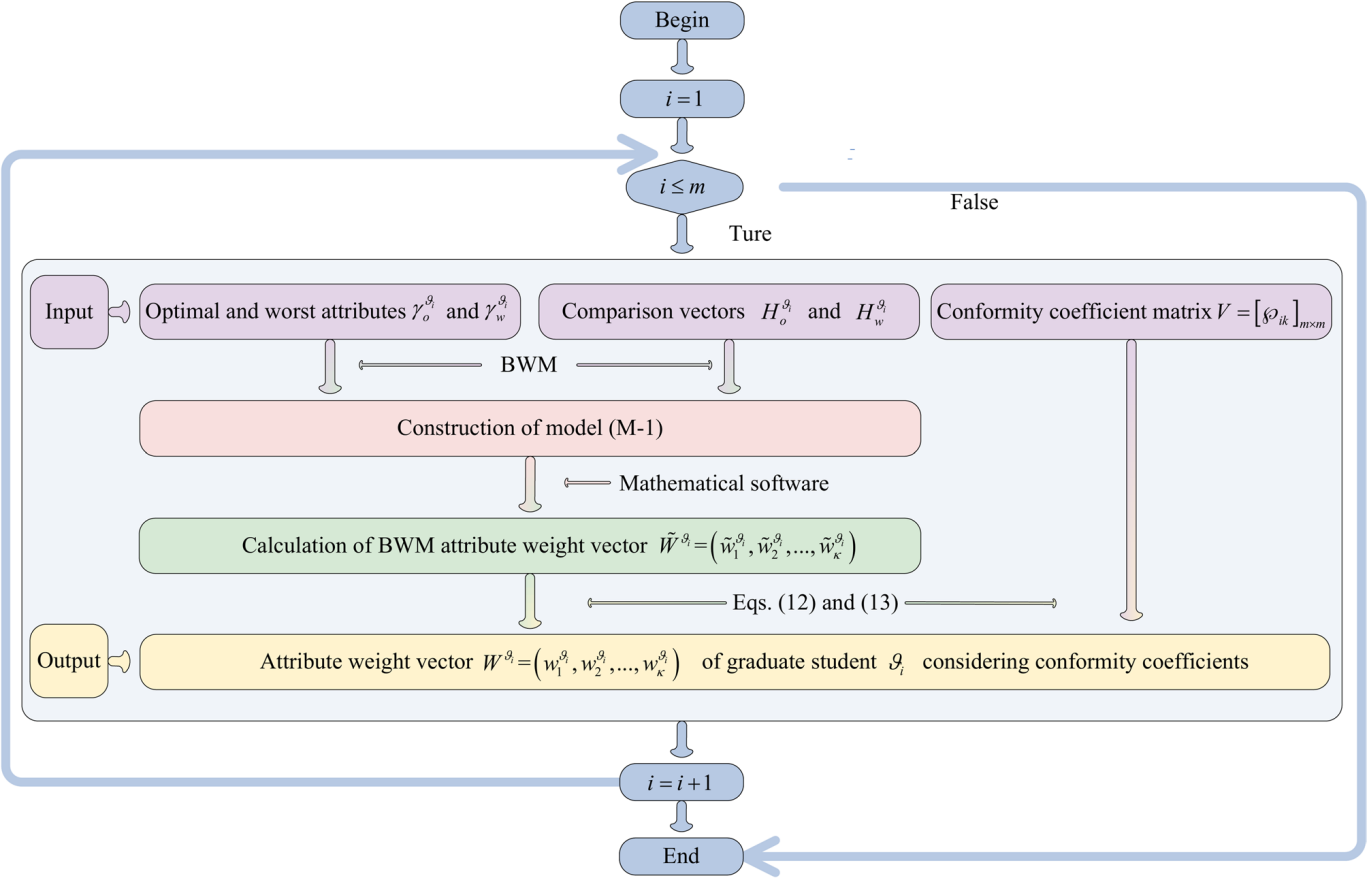
Flowchart of Algorithm 2.

To provide a more intuitive description of Algorithm 2, it is visually presented by Fig. 3.

#### Construction of the satisfaction matrix of graduate students for supervisors

Satisfaction matrix of graduate students for supervisors is set up by comprehensively using TOPSIS and grey correlation degrees. The specific processes are as follow:

First, the comprehensive PFM $G^{\vartheta } = \left[ {g_{ij}^{\vartheta } } \right]_{m \times n}$ of graduate students is constructed based on PFM $\Phi^{\vartheta } = \left[ {\phi_{itj}^{\vartheta } } \right]_{m \times \kappa \times n}$ and attribute weight vectors $W^{{\vartheta_{i} }} (i \in M)$.

14 $$g_{ij}^{\vartheta } = \sum\limits_{t = 1}^{\kappa } {\phi_{itj}^{\vartheta } } w_{t}^{{\vartheta_{i} }}$$

Second, positive ideal solution $G^{{\vartheta { + }}} = \left\{ {g_{1}^{{\vartheta { + }}} ,g_{2}^{{\vartheta { + }}} , \ldots ,g_{n}^{{\vartheta { + }}} } \right\}$ and negative ideal solution $G^{\vartheta - } = \left\{ {g_{1}^{\vartheta - } ,g_{2}^{\vartheta - } , \ldots ,g_{n}^{\vartheta - } } \right\}$ of graduate students are calculated based on comprehensive PFM $G^{\vartheta } = \left[ {g_{ij}^{\vartheta } } \right]_{m \times n}$; then distances $D(g_{ij}^{\vartheta } ,g^{\vartheta + } )$ and $D(g_{ij}^{\vartheta } ,g^{\vartheta - } )$ between evaluation value $g_{ij}^{\vartheta }$ and positive ideal solution $G^{{\vartheta { + }}}$ and negative ideal solution $G^{\vartheta - }$ are calculated using Eq. (4). On this basis, closeness matrix ${\rm Z}^{\vartheta } = \left[ {\xi_{{g_{ij}^{\vartheta } }} } \right]_{m \times n}$ is established, where $\xi_{{g_{ij}^{\vartheta } }}$ is calculated as:

15 $$\xi_{{g_{ij}^{\vartheta } }} = \frac{{D(g_{ij}^{\vartheta } ,g^{\vartheta - } )}}{{D(g_{ij}^{\vartheta } ,g^{\vartheta - } ) + D(g_{ij}^{\vartheta } ,g^{\vartheta + } )}}$$

In Eq. (15), $\xi_{{g_{ij}^{\vartheta } }}$ is the closeness between evaluation value $g_{ij}^{\vartheta }$ and positive ideal solution $g^{\vartheta + }$, and between evaluation value $g_{ij}^{\vartheta }$ and negative ideal solution $g^{\vartheta - }$. The larger the value of $\xi_{{g_{ij}^{\vartheta } }}$, the closer it is to the positive ideal solution and the farther it is from the negative ideal solution, indicating a higher satisfaction; on the contrary, $\xi_{{g_{ij}^{\vartheta } }}$ becomes more distant from the positive ideal solution and closer to the negative ideal solution, indicating a lower satisfaction.

Then, based on comprehensive PFM $G^{\vartheta } = \left[ {g_{ij}^{\vartheta } } \right]_{m \times n}$, positive and negative correlations ($Q_{ij}^{\vartheta + }$ and $Q_{ij}^{\vartheta - }$) between evaluation value $g_{ij}^{\vartheta }$ and positive and negative ideal solutions ($g_{ij}^{{\vartheta { + }}}$ and $g_{ij}^{\vartheta - }$) are calculated as:

16 $$Q_{ij}^{\vartheta + } = \frac{{\mathop {\min }\limits_{i \in M,j \in N} \left\{ {D(g_{ij}^{\vartheta } ,g_{j}^{\vartheta + } )} \right\} - \rho \mathop {\max }\limits_{i \in M,j \in N} \left\{ {D(g_{ij}^{\vartheta } ,g_{j}^{\vartheta + } )} \right\}}}{{D(g_{ij}^{\vartheta } ,g_{j}^{\vartheta + } ) - \rho \mathop {\max }\limits_{i \in M,j \in N} \left\{ {D(g_{ij}^{\vartheta } ,g_{j}^{\vartheta + } )} \right\}}}$$

17 $$Q_{ij}^{\vartheta - } = \frac{{\mathop {\min }\limits_{i \in M,j \in N} \left\{ {D(g_{ij}^{\vartheta } ,g_{j}^{\vartheta - } )} \right\} - \rho \mathop {\max }\limits_{i \in M,j \in N} \left\{ {D(g_{ij}^{\vartheta } ,g_{j}^{\vartheta - } )} \right\}}}{{D(g_{ij}^{\vartheta } ,g_{j}^{\vartheta - } ) - \rho \mathop {\max }\limits_{i \in M,j \in N} \left\{ {D(g_{ij}^{\vartheta } ,g_{j}^{\vartheta - } )} \right\}}}$$

In Eqs. (16) and (17), $\rho$ is the distinguishing coefficient used to adjust the proportion of $\mathop {\max }\limits_{i \in M,j \in N} \left\{ {D(g_{ij}^{\vartheta } ,g_{i}^{\vartheta - } )} \right\}$ and $D(g_{ij}^{\vartheta } ,g_{i}^{\vartheta - } )$ in grey correlation degrees calculation., $\rho$ is usually taken as 0.5, $\rho \in (0,1)$.

Based on positive and negative correlations $Q_{ij}^{\vartheta + }$ and $Q_{ij}^{\vartheta - }$, grey correlation degree matrix 
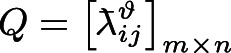
$Q = \left[ { \texttt{ƛ}_{ij}^{\vartheta } } \right]_{m \times n}$ is built, where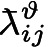 is represented as:18

Furthermore, satisfaction matrix $A = \left[ {a_{ij} } \right]_{m \times n}$ of graduate students for supervisors is set up based on the closeness matrix ${\rm Z}^{\vartheta } = \left[ {\xi_{{g_{ij}^{\vartheta } }} } \right]_{m \times n}$ and grey correlation degree matrix 
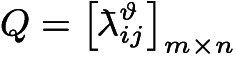
, where $a_{ij}$ is computed as:19

In Eq. (19), $\tau$ is the grey correlation coefficient, which is used to adjust the proportion of grey correlation degree and closeness, satisfying $0 \le \tau \le 1$.

In summary, an algorithm for constructing satisfaction matrix $A = \left[ {a_{ij} } \right]_{m \times n}$ of graduate students for supervisors is developed and its pseudocode is displayed as follows:

**Algorithm 3:**
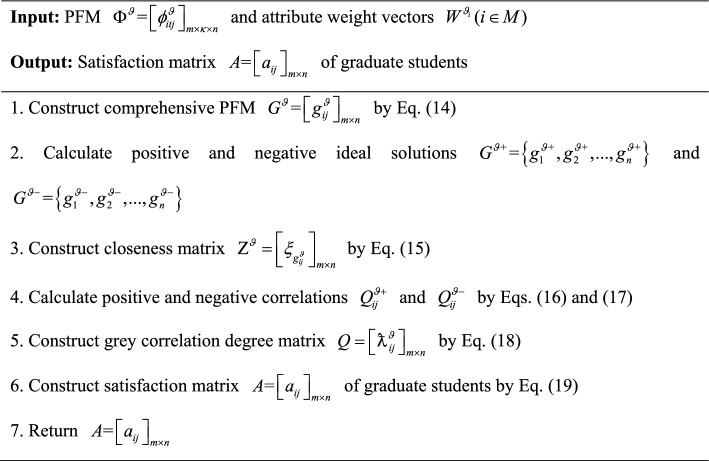
Construction of satisfaction matrix $A{\text = }\left[ {a_{{ij}} } \right]_{{m \times n}}$ of graduate students

**Fig. 4:**
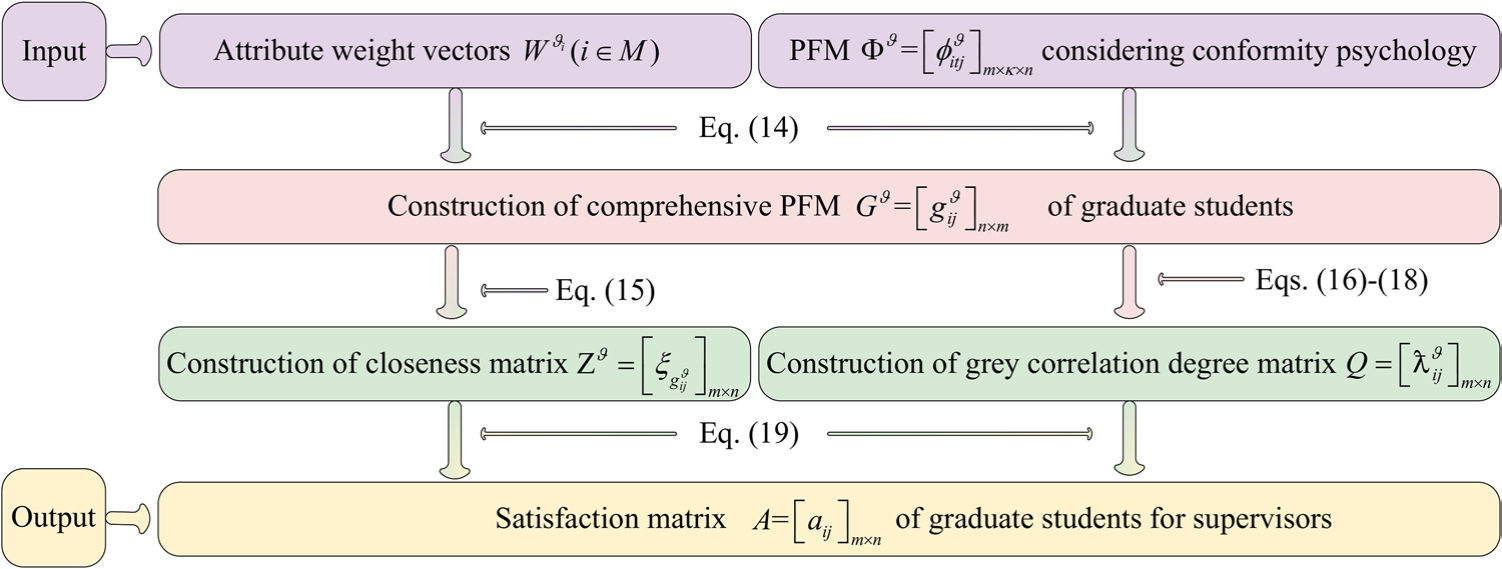
Flowchart of Algorithm 3.

To provide a more intuitive description of Algorithm 3, it is visually presented by Fig. 4.

### Construction of satisfaction matrix of supervisors for graduate students

#### Construction of Pythagorean fuzzy matrix considering graduate students’ preferences

In this subsection, preference coefficient matrix $U = \left[ {\mu_{ij}^{\vartheta } } \right]_{m \times n}$ of graduate students is calculated using TODIM based on comprehensive PFM $G^{\vartheta } = \left[ {g_{ij}^{\vartheta } } \right]_{n \times m}$. Then, PFM $\Phi^{\chi } = \left[ {\phi_{jli}^{\chi } } \right]_{n \times p \times m}$ considering graduate students’ preferences is constructed. The specific procedure is as follows:

First, PFM $\Psi^{\chi } = \left[ {\varphi_{jli}^{\chi } } \right]_{n \times p \times m}$ of graduate students is established using Eq. (5) based on LTM $S^{\chi } = \left[ {s_{jli}^{\chi } } \right]_{n \times p \times m}$.

Second, dominance degree $\Psi_{i}^{\vartheta } \left( {\psi_{j} ,\psi_{h} } \right)$ of graduate student $\vartheta_{i}$ selecting supervisor $\chi_{j}$, relative to supervisor $\chi_{h}$, is determined based on comprehensive PFM $G^{\vartheta } = \left[ {g_{ij}^{\vartheta } } \right]_{n \times m}$ and is expressed as:

20 $$\Psi_{i}^{\vartheta } \left( {\psi_{j} ,\psi_{h} } \right) = \left\{ \begin{gathered} d\left( {g_{ij}^{\vartheta } ,g_{ih}^{\vartheta } } \right),g_{ij}^{\vartheta } \ge g_{ih}^{\vartheta } \hfill \\ - \frac{1}{\theta }d\left( {g_{ij}^{\vartheta } ,g_{ih}^{\vartheta } } \right),g_{ij}^{\vartheta } < g_{ih}^{\vartheta } \hfill \\ \end{gathered} \right.$$

In Eq. (20), $\theta$ ($\theta$ > 0) is the loss attenuation coefficient, indicating the degree of loss avoidance of graduate students. The smaller $\theta$, the greater the degree of loss avoidance.

Based on dominance degrees $$\Psi_{i}^{\vartheta } \left( {\psi_{j} ,\psi_{h} } \right)$$$$(h \in N)$$, overall dominance degree $$\delta_{ij}^{\vartheta }$$ of graduate student $$\vartheta_{i}$$ selecting supervisor $$\chi_{j}$$ is calculated as:

21 $$\delta_{ij}^{\vartheta } = \sum\limits_{h = 1}^{n} {\Psi_{i}^{\vartheta } \left( {\psi_{j} ,\psi_{h} } \right)}$$

Based on overall dominance degrees $$\delta_{ij}^{\vartheta }$$$$(i \in M,j \in N)$$, preference coefficient matrix $$U = \left[ {\mu_{ij}^{\vartheta } } \right]_{m \times n}$$ of graduate students is built, where $$\mu_{ij}^{\vartheta }$$ is displayed as:

22 $$\mu_{ij}^{\vartheta } = \frac{{\delta_{ij}^{\vartheta } - \mathop {\min }\limits_{i \in M,j \in N} \left\{ {\delta_{ij}^{\vartheta } } \right\}}}{{\mathop {\max }\limits_{i \in M,j \in N} \left\{ {\delta_{ij}^{\vartheta } } \right\} - \mathop {\min }\limits_{i \in M,j \in N} \left\{ {\delta_{ij}^{\vartheta } } \right\}}}$$

Furthermore, PFM $\Phi^{\chi } = \left[ {\phi_{jli}^{\chi } } \right]_{n \times p \times m}$ considering graduate students’ preferences is set up based on PFM $\Psi^{\chi } = \left[ {\varphi_{jli}^{\chi } } \right]_{n \times p \times m}$ and preference coefficient matrix $U = \left[ {\mu_{ij}^{\vartheta } } \right]_{m \times n}$, where $\phi_{jli}^{\chi }$ is computed as:

23 $$\phi_{jli}^{\chi } = \left( {\varphi_{jli}^{\chi } } \right)^{\beta } \mathop \Pi \limits_{f} \left( {\varphi_{fli}^{\chi } } \right)^{{\left( {1 - \beta } \right)\mu_{if}^{\vartheta } }} = \left\{ {\left( {\left( {u_{jli}^{\chi } } \right)^{\beta } \left( {\mathop \Pi \limits_{{f:u_{fli}^{{\chi_{j} }} \ne 0}} \left( {u_{fli}^{\chi } } \right)^{{\mu_{if}^{\vartheta } }} } \right)^{{\left( {1 - \beta } \right)}} ,\left( {v_{jli}^{\chi } } \right)^{\beta } \left( {\mathop \Pi \limits_{{f:v_{fli}^{{\chi_{j} }} \ne 0}} \left( {v_{fli}^{\chi } } \right)^{{\mu_{if}^{\vartheta } }} } \right)^{{\left( {1 - \beta } \right)}} } \right)} \right.$$

In Eq. (23), $\beta$ is the decision adjustment coefficient, which is used to adjust the proportion of supervisors’ internal decisions and others decisions that consider graduate students’ preferences, satisfying $0 \le \beta \le 1$.

*Note 3* In the actual GSSM problem, attributes considered by supervisors in this paper include academic performance, English proficiency, academic experience and learning autonomy. All these attributes are categorized as benefit attributes. Therefore, the solution process outlined above does not incorporate methods for handling cost attributes. If cost attributes exist in practical scenarios, PFM $\Phi^{\chi } = \left[ {\phi_{jli}^{\chi } } \right]_{n \times p \times m}$ can be converted into the BPF matrix $\Phi^{\prime \chi } = \left[ {\phi_{jli}^{\prime \chi } } \right]_{n \times p \times m}$ using Eq. (24), where $\phi_{jli}^{\prime \chi }$ is computed as:

24 $$\phi_{jli}^{\prime \chi } = \left\{ \begin{gathered} \phi_{jli}^{\chi } ,{\text{ benefit attribute }} \hfill \\ \overline{\phi }_{jli}^{\chi } ,{\text{ cost attribute}} \hfill \\ \end{gathered} \right.$$

In Eq. (24), when attribute $\chi_{l}$ is the benefit attribute, the BPF number corresponding to $\phi_{jli}^{\chi }$ is itself. When attribute $\chi_{l}$ is the cost one, the complement of BPF number corresponding to $\phi_{jli}^{\chi }$ is $\overline{\phi }_{jli}^{\chi }$.

In summary, an algorithm for constructing PFM $\Phi^{\chi } = \left[ {\phi_{jli}^{\chi } } \right]_{n \times p \times m}$ considering graduate students’ preferences is developed and its pseudocode is displayed as follows:

**Algorithm 4:**
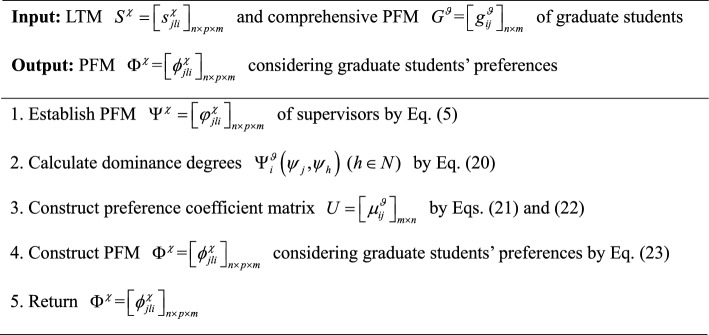
Construction of PFM $\Phi ^{\chi } {\text = }\left[ {\phi _{{jli}}^{\chi } } \right]_{{n \times p \times m}}$ considering graduate students’ preferences

**Fig. 5:**
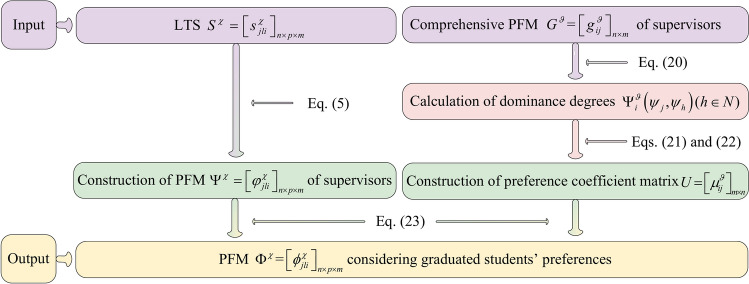
Flowchart of Algorithm 4.

To provide a more intuitive description of Algorithm 4, it is visually presented by Fig. 5.

#### Calculation of attribute weight vectors of supervisors

In this subsection, attribute weight vector $W^{{\chi_{j} }} = \left( {w_{1}^{{\chi_{j} }} ,w_{2}^{{\chi_{j} }} , \ldots ,w_{p}^{{\chi_{j} }} } \right)$ of supervisor $\chi_{j}$ is determined using the BWM method. The solving procedure is as follows:

First, the optimal attribute $\gamma_{o}^{{\chi_{j} }}$ and the worst attribute $\gamma_{w}^{{\chi_{j} }}$ of attribute set $\gamma^{\chi }$ are selected by supervisor $\chi_{j}$; Then comparison vector $H_{o}^{{\chi_{j} }} = \left( {h_{o1}^{{\chi_{j} }} ,h_{o2}^{{\chi_{j} }} , \ldots ,h_{op}^{{\chi_{j} }} } \right)$ of the optimal attribute $\gamma_{o}^{{\chi_{j} }}$ compared to other attributes $\gamma_{l}^{{\chi_{j} }} (l = 1,2, \ldots ,p;l \ne o)$ is constructed by supervisor $\chi_{j}$ using the 1–9 scale, and comparison vector $H_{w}^{{\chi_{j} }} = \left( {h_{1w}^{{\chi_{j} }} ,h_{2w}^{{\chi_{j} }} , \ldots ,h_{pw}^{{\chi_{j} }} } \right)$ of other attributes $\gamma_{l}^{{\chi_{j} }} (l = 1,2,...,p;l \ne o)$ compared to the worst attribute $\gamma_{w}^{{\chi_{j} }}$ is constructed by supervisor $\chi_{j}$ using the 1–9 scale.

Second, the following mathematical programming model (M-2) is constructed based on information given by supervisor $\chi_{j}$:

$$(M - 2)\left\{ \begin{gathered} Min\delta \hfill \\ s.t.\left| {\frac{{w_{o}^{{\chi_{j} }} }}{{w_{h}^{{\chi_{j} }} }} - h_{oh}^{{\chi_{j} }} } \right| \le \iota ,h = 1,2, \ldots ,p; \hfill \\ \left| {\frac{{w_{h}^{{\chi_{j} }} }}{{w_{w}^{{\chi_{j} }} }} - h_{hw}^{{\chi_{j} }} } \right| \le \iota ,h = 1,2, \ldots ,p; \hfill \\ \sum\limits_{h = 1}^{p} {w_{h}^{{\chi_{j} }} } = 1,w_{h}^{{\chi_{j} }} \ge 0,h = 1,2, \ldots ,p. \hfill \\ \end{gathered} \right.$$

Finally, the weight vector $W^{{\chi_{j} }} = \left( {w_{1}^{{\chi_{j} }} ,w_{2}^{{\chi_{j} }} , \ldots ,w_{p}^{{\chi_{j} }} } \right)$ of supervisor $\chi_{j}$ for attribute set $\gamma^{\vartheta }$ is determined by solving model (M-2) using mathematical software.

In summary, an algorithm for calculating attribute weight vectors $W^{{\chi_{j} }} \left( {j \in N} \right)$ of supervisors is developed and its pseudocode is displayed as follows:

**Algorithm 5:**
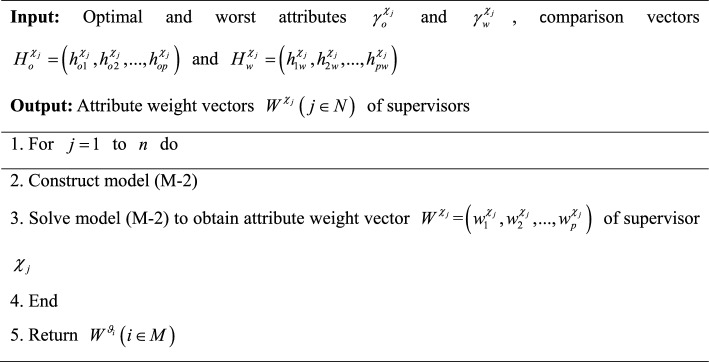
Calculation of attribute weight vectors $W^{{\chi _{j} }} \left( {j \in N} \right)$ of supervisors

**Fig. 6:**
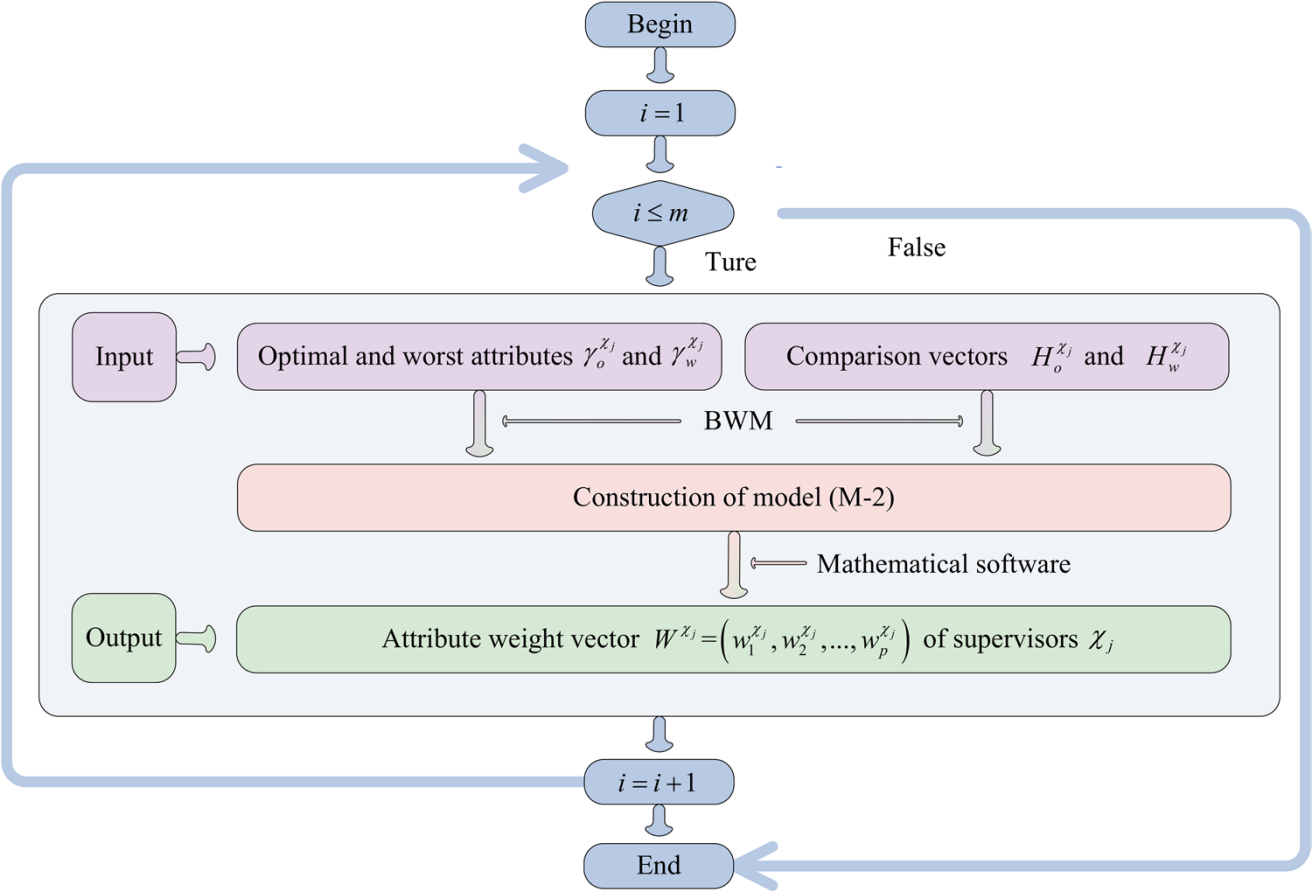
Flowchart of Algorithm 5.

To provide a more intuitive description of Algorithm 5, it is visually presented by Fig. 6.

#### Construction of satisfaction matrix of supervisors for graduate students

In this subsection, satisfaction matrix $B = \left[ {b_{ij} } \right]_{m \times n}$ of supervisors for graduate students is set up using TOPSIS and grey correlation degrees based on comprehensive PFM $G^{\chi } = \left[ {g_{ij}^{\chi } } \right]_{m \times n}$. The specific procedure is as follows:

First, comprehensive PFM $G^{\chi } = \left[ {g_{ij}^{\chi } } \right]_{m \times n}$ of supervisors is built based on PFM $\Phi^{\chi } = \left[ {\phi_{jli}^{\chi } } \right]_{n \times p \times m}$ and attribute weight vectors $W^{{\chi_{j} }} (j \in N)$, where $g_{ij}^{\chi }$ is represented as:

25 $$g_{ij}^{\chi } = \sum\limits_{l = 1}^{p} {\phi_{ilj}^{\chi } } w_{t}^{{\chi_{j} }}$$

Second, positive and negative ideal solutions $G^{{\chi { + }}} = \left\{ {g_{1}^{{\chi { + }}} ,g_{2}^{{\chi { + }}} ,...,g_{n}^{{\chi { + }}} } \right\}$ and $G^{\chi - } = \left\{ {g_{1}^{\chi - } ,g_{2}^{\chi - } ,...,g_{n}^{\chi - } } \right\}$ of supervisors are determined using Eq. (4) based on comprehensive PFM $G^{\chi } = \left[ {g_{ij}^{\chi } } \right]_{m \times n}$; distances $D(g_{ij}^{\chi } ,g^{\chi + } )$ and $D(g_{ij}^{\chi } ,g^{\chi - } )$ between $g_{ij}^{\chi }$ and positive and negative ideal solutions ($g^{\chi + }$ and $g^{\chi - }$) are calculated, then closeness matrix ${\rm Z}^{\chi } = \left[ {\xi_{{g_{ij}^{\chi } }} } \right]_{m \times n}$ is constructed, where $\xi_{{g_{ij}^{\chi } }}$ is presented as:

26 $$\xi_{{g_{ij}^{\chi } }} = \frac{{D(g_{ij}^{\chi } ,g^{\chi - } )}}{{D(g_{ij}^{\chi } ,g^{\chi - } ) + D(g_{ij}^{\chi } ,g^{\chi + } )}}$$

In Eq. (26), the larger the value of $\xi_{{g_{ij}^{\chi } }}$, the closer it is to the positive ideal solution and the farther it is from the negative ideal solution, indicating a higher satisfaction, the reverse is true.

Based on comprehensive PFM $$G^{\chi } = \left[ {g_{ij}^{\chi } } \right]_{m \times n}$$, positive and negative correlations $$Q_{ij}^{\chi + }$$$$(i \in M,j \in N)$$ and $$Q_{ij}^{\chi - }$$$$(i \in M,j \in N)$$ between evaluation value $$g_{ij}^{\chi }$$ and positive and negative ideal solutions ($$g^{\chi + }$$ and $$g^{\chi - }$$) are calculated as:

27 $$Q_{ij}^{\chi + } = \frac{{\mathop {\min }\limits_{i \in M,j \in N} \left\{ {D(g_{ij}^{\chi } ,g_{i}^{\chi + } )} \right\} - \rho \mathop {\max }\limits_{i \in M,j \in N} \left\{ {D(g_{ij}^{\chi } ,g_{i}^{\chi + } )} \right\}}}{{D(g_{ij}^{\chi } ,g_{i}^{\chi + } ) - \rho \mathop {\max }\limits_{i \in M,j \in N} \left\{ {D(g_{ij}^{\chi } ,g_{i}^{\chi + } )} \right\}}}$$

28 $$Q_{ij}^{\chi - } = \frac{{\mathop {\min }\limits_{i \in M,j \in N} \left\{ {D(g_{ij}^{\chi } ,g_{i}^{\chi - } )} \right\} - \rho \mathop {\max }\limits_{i \in M,j \in N} \left\{ {D(g_{ij}^{\chi } ,g_{i}^{\chi - } )} \right\}}}{{D(g_{ij}^{\chi } ,g_{i}^{\chi - } ) - \rho \mathop {\max }\limits_{i \in M,j \in N} \left\{ {D(g_{ij}^{\chi } ,g_{i}^{\chi - } )} \right\}}}$$

In Eqs. (27) and (28), $\rho$ is the resolution coefficient used to adjust the proportion of $\mathop {\max }\limits_{i \in M,j \in N} \left\{ {D(g_{ij}^{\chi } ,g_{i}^{\chi - } )} \right\}$ and $D(g_{ij}^{\chi } ,g_{i}^{\chi - } )$ in the calculation of grey correlation degrees, where $\rho \in (0,1)$ and $\rho$ usually taken as 0.5.

Based on positive and negative correlations $$Q_{ij}^{\chi + }$$$$(i \in M,j \in N)$$ and $$Q_{ij}^{\chi - }$$$$(i \in M,j \in N)$$, grey correlation degree matrix 
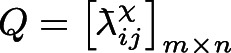
 is constructed, where 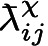 is represented as:29

Furthermore, satisfaction matrix $B = \left[ {b_{ij} } \right]_{m \times n}$ of supervisors is constructed based on closeness matrix ${\rm Z}^{\chi } = \left[ {\xi_{{g_{ij}^{\chi } }} } \right]_{m \times n}$ and grey correlation degree matrix 
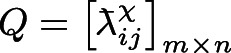
, where $b_{ij}$ is computed as:30

In Eq. (30), $\tau$ is the grey correlation coefficient used to adjust the proportion of grey correlation degrees and closeness, $0 \le \tau \le 1$.

In summary, the algorithm for constructing satisfaction matrix $B = \left[ {b_{ij} } \right]_{m \times n}$ of supervisors for graduate students is as follows:

In summary, an algorithm for constructing satisfaction matrix $B = \left[ {b_{ij} } \right]_{m \times n}$ of supervisors is developed and its pseudocode is displayed as follows:

**Algorithm 6:**
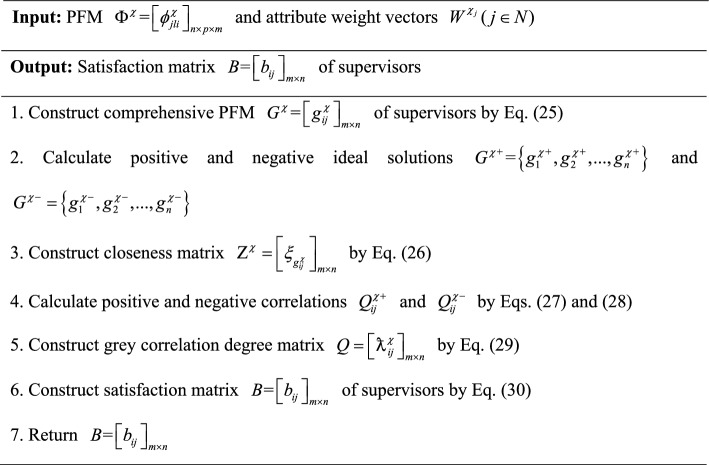
Construction of satisfaction matrix $B = \left[ {b_{{ij}} } \right]_{{m \times n}}$ of supervisors

**Fig. 7:**
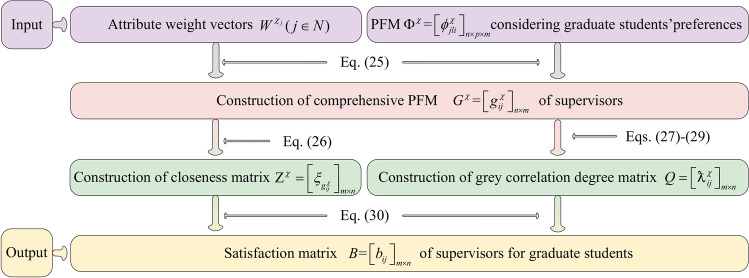
Flowchart of Algorithm 6.

To provide a more intuitive description of Algorithm 6, it is visually presented by Fig. 7.

### Establishment of a many-to-one matching model considering stability-based fairness

First, matching matrix $X = \left[ {x_{ij} } \right]_{m \times n}$ and stable matching matrix $E = \left[ {\sigma_{ij}^{\Upsilon } } \right]_{m \times n}$ are introduced, where $$x_{ij} = \left\{ {\begin{array}{*{20}c} {1,} & {\varphi (\chi_{j} ) = \vartheta_{i} } \\ {0,} & {\varphi (\chi_{j} ) \ne \vartheta_{i} } \\ \end{array} } \right.$$, $$\sigma_{ij}^{\Upsilon } = \left\{ {\begin{array}{*{20}c} {x_{ij} ,} & {x_{ij} + \sum\limits_{{k:\tilde{a}_{is} \ge \tilde{a}_{ij} }} {x_{is} } + \sum\limits_{{k:\tilde{b}_{lj} \ge \tilde{b}_{ij} }} {x_{lj} } \ge 1} \\ {0,} & {{\text{else}}} \\ \end{array} } \right.$$. According to satisfaction matrices $A = \left[ {a_{ij} } \right]_{m \times n}$ and $B = \left[ {b_{ij} } \right]_{m \times n}$, matching matrix $X = \left[ {x_{ij} } \right]_{m \times n}$ and stable matching matrix $E = \left[ {\sigma_{ij}^{\Upsilon } } \right]_{m \times n}$, the following many-to-one GSSM model (M-3) is constructed as:

$$\left( {\text{M - 3}} \right)\left\{ \begin{gathered} {\text{Max}}\;D_{1} = \sum\limits_{i = 1}^{m} {\sum\limits_{j = 1}^{n} {a_{ij} } } x_{ij} ; \hfill \\ {\text{Max}}\;D_{2} = \sum\limits_{i = 1}^{m} {\sum\limits_{j = 1}^{n} {b_{ij} x_{ij} } } \hfill \\ {\text{Min}}\;D_{3} = \sum\limits_{i = 1}^{m} {\sum\limits_{j = 1}^{n} {\frac{{\mathop {\max }\limits_{i \in M,j \in N} (a_{ij} - b_{ij} )^{2} - (a_{ij} - b_{ij} )^{2} }}{{\mathop {\max }\limits_{i \in M,j \in N} (a_{ij} - b_{ij} )^{2} - \mathop {\min }\limits_{i \in M,j \in N} (a_{ij} - b_{ij} )^{2} }}\sigma_{ij}^{\Upsilon } } } ; \hfill \\ {\text{s}}{\text{.t}}{.}\;\sum\limits_{j = 1}^{n} {x_{ij} } = 1,i \in M; \hfill \\ 1 \le \sum\limits_{i = 1}^{m} {x_{ij} } \le c_{j} ,j \in N; \hfill \\ x_{ij} \in \left\{ {0,1} \right\},i \in M,j \in N. \hfill \\ \end{gathered} \right.$$

In model (M-3), ${\text{Max}}\;D_{1}$ represents maximizing the sum of graduate students’ satisfactions, ${\text{Max}}\;D_{2}$ represents maximizing the sum of supervisors’ satisfactions, and ${\text{Max }}D_{3}$ represents maximizing the stability-based fairness; $1 \le \sum\limits_{i = 1}^{m} {x_{ij} } \le c_{j}$ represents the matching quantity constraint of supervisor $\chi_{j}$, and $\sum\limits_{j = 1}^{n} {x_{ij} } = 1$ represents the matching quantity constraint of graduate student $\vartheta_{i}$.

### Establishment of a one-to-one matching model considering stability-based fairness

To solve model (M-3), the many-to-one bilateral matching model (M-3) is transformed to one-to-one matching model by introducing virtual subjects. On the one hand, in view of the maximum matching number of supervisor $\chi_{j}$ is $c_{j}$, $c_{j}$ virtual supervisors with the same preferences are introduced, denoted as $\chi_{j}^{1} ,\chi_{j}^{2} , \ldots ,\chi_{j}^{{c_{j} }}$, where matching number of $\chi_{j}^{o} (o = 1,2, \ldots ,c_{j} )$ is 1. Therefore, original supervisor set $\chi$ is converted into a virtual supervisor set $\tilde{\chi } = \left\{ {\chi_{1}^{1} ,\chi_{1}^{2} , \ldots ,\chi_{1}^{{c_{1} }} ,\chi_{2}^{1} ,\chi_{2}^{2} , \ldots ,\chi_{2}^{{c_{2} }} , \ldots ,\chi_{n}^{1} ,\chi_{n}^{2} , \ldots ,\chi_{n}^{{c_{n} }} } \right\}$, abbreviated as $\tilde{\chi } = \left\{ {\tilde{\chi }_{1} ,\tilde{\chi }_{2} , \ldots ,\tilde{\chi }_{r} } \right\}$, where $r = \sum\limits_{j = 1}^{n} {c_{j} }$. Then, based on satisfaction matrix $B = \left[ {b_{ij} } \right]_{m \times n}$ of supervisors for graduate students, an extended satisfaction matrix $\tilde{B} = \left[ {\tilde{b}_{ig} } \right]_{m \times r}$ of virtual supervisors for graduate students is constructed, where the extended satisfaction $\tilde{b}_{ig}$ of virtual supervisor $\tilde{\chi }_{g}$ for graduate student $\vartheta_{i}$ is represented as:

31 $$\tilde{b}_{ig} = \left\{ \begin{gathered} b_{i1} ,1 \le g \le c_{1} \hfill \\ b_{i2} ,c_{1} + 1 \le g \le c_{1} + c_{2} \hfill \\ ... \hfill \\ b_{in} ,c_{1} + ... + c_{n - 1} + 1 \le g \le r \hfill \\ \end{gathered} \right.$$

On the other hand, based on satisfaction matrix $A = \left[ {a_{ij} } \right]_{m \times n}$ of graduate students for supervisors, an extended satisfaction matrix $\tilde{A} = \left[ {\tilde{a}_{ig} } \right]_{m \times d}$ of graduate students for virtual supervisors is constructed, where extended satisfaction $\tilde{a}_{ig}$ of graduate student $\vartheta_{i}$ for virtual supervisor $\tilde{\chi }_{g}$ is represented as

32 $$\tilde{a}_{ig} = \left\{ \begin{gathered} a_{i1} ,\begin{array}{*{20}c} {} & {} & {} & {1 \le g \le c_{1} } \\ \end{array} \hfill \\ a_{i2} ,\begin{array}{*{20}c} {} & {} & {c_{1} + 1 \le g \le c_{1} + c_{2} } \\ \end{array} \hfill \\ ...\begin{array}{*{20}c} {} & {} & {...} \\ \end{array} \hfill \\ a_{in} ,\begin{array}{*{20}c} {} & {c_{1} + ... + c_{n - 1} + 1 \le g \le r} \\ \end{array} \hfill \\ \end{gathered} \right.$$

Furthermore, based on satisfaction matrices $\tilde{A} = \left[ {\tilde{a}_{ig} } \right]_{m \times d}$ and $\tilde{B} = \left[ {\tilde{b}_{ig} } \right]_{m \times r}$, a matching matrix $X = \left[ {x_{ig} } \right]_{m \times r}$ and a stable matching matrix $E = \left[ {\sigma_{ig}^{\Upsilon } } \right]_{m \times r}$ are introduced, where $$x_{ig} = \left\{ {\begin{array}{*{20}c} {1,} & {\varphi (\tilde{\chi }_{g} ) = \vartheta_{i} } \\ {0,} & {\varphi (\tilde{\chi }_{g} ) \ne \vartheta_{i} } \\ \end{array} } \right.$$ and $$\sigma_{ig}^{\Upsilon } = \left\{ {\begin{array}{*{20}c} {x_{ig} ,} & {x_{ig} + \sum\limits_{{k:\tilde{a}_{is} \ge \tilde{a}_{ig} }} {x_{is} } + \sum\limits_{{k:\tilde{b}_{lg} \ge \tilde{b}_{ig} }} {x_{lg} } \ge 1} \\ {0,} & {{\text{else}}} \\ \end{array} } \right.$$. Then, the following one-on-one matching model (M-4) between GSS is constructed:

$$\left( {M - 4} \right)\left\{ \begin{gathered} Max\;D_{4} = \sum\limits_{i = 1}^{m} {\sum\limits_{g = 1}^{r} {\tilde{a}_{ig} } } x_{ig} ; \hfill \\ Max\;D_{5} = \sum\limits_{i = 1}^{m} {\sum\limits_{g = 1}^{r} {\tilde{b}_{ig} x_{ig} ;} } \hfill \\ Max\;D_{6} = \sum\limits_{i = 1}^{m} {\sum\limits_{g = 1}^{r} {\frac{{\mathop {\max }\limits_{g \in R,i \in M} (\tilde{a}_{ig} - \tilde{b}_{ig} )^{2} - (\tilde{a}_{ig} - \tilde{b}_{ig} )^{2} }}{{\mathop {\max }\limits_{g \in R,i \in M} (\tilde{a}_{ig} - \tilde{b}_{ig} )^{2} - \mathop {\min }\limits_{g \in R,i \in M} (\tilde{a}_{ig} - \tilde{b}_{ig} )^{2} }}\sigma_{ig}^{\Upsilon } } } ; \hfill \\ s.t.\;\sum\limits_{g = 1}^{r} {x_{ig} } = 1,i \in M; \hfill \\ \sum\limits_{i = 1}^{m} {x_{ig} } \le 1,g \in R; \hfill \\ x_{ig} \in \left\{ {0,1} \right\},g \in R,i \in M. \hfill \\ \end{gathered} \right.$$

In model (M-4), ${\text{Max}}\;D_{4}$ represents maximizing the sum of graduate students’ satisfactions, ${\text{Max}}\;D_{5}$ represents maximizing the sum of supervisors’ satisfactions, and ${\text{Max }}D_{6}$ represents maximizing the stability-based fairness; $\sum\limits_{i = 1}^{m} {x_{ig} } \le 1$ indicates the matching quantity constraint of supervisors; and $\sum\limits_{g = 1}^{r} {x_{ig} } = 1$ indicates the matching quantity constraint of graduate students.

### Solution of the one-to-one matching model considering stability-based fairness

The model (M-4) is transformed into the following single objective model (M-5) using linear weighting method:

$$\left( {M - 5} \right)\left\{ \begin{gathered} Max\;D = \sum\limits_{g = 1}^{r} {\sum\limits_{j = 1}^{n} {\left( {\omega_{1} \tilde{a}_{ig} x_{ig} + \omega_{2} \tilde{b}_{ig} x_{ig} + \omega_{3} \frac{{\mathop {\max }\limits_{g \in R,i \in M} (\tilde{a}_{ig} - \tilde{b}_{ig} )^{2} - (\tilde{a}_{ig} - \tilde{b}_{ig} )^{2} }}{{\mathop {\max }\limits_{g \in R,i \in M} (\tilde{a}_{ig} - \tilde{b}_{ig} )^{2} - \mathop {\min }\limits_{g \in R,i \in M} (\tilde{a}_{ig} - \tilde{b}_{ig} )^{2} }}\sigma_{ig}^{\Upsilon } } \right)} } ; \hfill \\ s.t.\;\sum\limits_{g = 1}^{r} {x_{ig} } = 1,i \in M; \hfill \\ \sum\limits_{i = 1}^{m} {x_{ig} } \le 1,g \in R; \hfill \\ x_{ig} \in \left\{ {0,1} \right\},g \in R,i \in M. \hfill \\ \end{gathered} \right.$$

In model (M-5), $\omega_{1}$, $\omega_{2}$ and $\omega_{3}$ are respectively weights of objective functions $D_{4}$, $D_{5}$ and $D_{6}$, satisfying $\omega_{1} ,\omega_{2} ,\omega_{3} \ge 0$ and $\omega_{1} { + }\omega_{2} { + }\omega_{3} = 1$. model (M-5) is solved using mathematical software such as Lingo to obtain the optimal stable matching scheme $\Upsilon^{ * }$.

### Matching decision-making process between graduate students and supervisors considering conformity psychology and graduate students’ preferences

In summary, decision-making steps for many-to-one matching between GSS considering conformity psychology and graduate students’ preferences are as follows:

*Step 1* Based on LTM $S^{\vartheta } = \left[ {s_{itj}^{\vartheta } } \right]_{m \times \kappa \times n}$ provided by graduate students, PFM $\Psi^{\vartheta } = \left[ {\varphi_{itj}^{\vartheta } } \right]_{m \times \kappa \times n}$ of graduate students is constructed using Eq. (5).

*Step 2* Based on graduate students’ transference relation, conformity coefficient matrix $V = \left[ {\wp_{ik} } \right]_{m \times m}$ of graduate students is built using Eqs. (7)-(9);

*Step 3* Based on PFM $\Psi^{\vartheta } = \left[ {\varphi_{itj}^{\vartheta } } \right]_{m \times \kappa \times n}$ and conformity coefficient matrix $V = \left[ {\wp_{ik} } \right]_{m \times m}$ of graduate students, PFM $\Phi^{\vartheta } = \left[ {\phi_{itj}^{\vartheta } } \right]_{m \times \kappa \times n}$ considering conformity psychology is constructed using Eq. (10).

*Step 4* Based on attribute comparison information provided by graduate students and conformity coefficient matrix $\Phi^{\vartheta } = \left[ {\phi_{itj}^{\vartheta } } \right]_{m \times \kappa \times n}$, attribute weight vectors $W^{{\vartheta_{i} }} (i \in M)$ considering conformity coefficients are determined using Algorithm 2.

*Step 5* Based on PFM $\Phi^{\vartheta } = \left[ {\phi_{ijt}^{\vartheta } } \right]_{n \times m \times \kappa }$ and attribute weight vectors $W^{{\vartheta_{i} }} (i \in M)$, comprehensive PFM $G^{\vartheta } = \left[ {g_{ij}^{\vartheta } } \right]_{n \times m}$ of graduate students is set up using Eq. (13). Then satisfaction matrix $A = \left[ {a_{ij} } \right]_{m \times n}$ of graduate students for supervisors is constructed using Eqs. (14)-(18).

*Step 6* Based on LTM $S^{\chi } = \left[ {s_{jli}^{\chi } } \right]_{n \times p \times m}$ provided by supervisors, PFM $\Psi^{\chi } = \left[ {\varphi_{jli}^{\chi } } \right]_{n \times p \times m}$ of supervisors is constructed using Eq. (5).

*Step 7* Based on comprehensive PFM $G^{\vartheta } = \left[ {g_{ij}^{\vartheta } } \right]_{n \times m}$ of graduate students, preference coefficient matrix $U = \left[ {\mu_{ij}^{\vartheta } } \right]_{m \times n}$ of graduate students is constructed using Eqs. (19)-(21);

*Step 8* Based on PFM $\Psi^{\chi } = \left[ {\varphi_{jli}^{\chi } } \right]_{n \times p \times m}$ and preference coefficient matrix $U = \left[ {\mu_{ij}^{\vartheta } } \right]_{m \times n}$, PFM $\Phi^{\chi } = \left[ {\phi_{jli}^{\chi } } \right]_{n \times p \times m}$ considering graduate students’ preferences is built using Eq. (22).

*Step 9* Based on attribute comparison information provided by supervisors, attribute weight vectors $W^{{\chi_{j} }} (j \in N)$ of supervisors are determined using Algorithm 5.

*Step 10* Based on PFM $\Phi^{\chi } = \left[ {\phi_{jli}^{\chi } } \right]_{n \times p \times m}$ and attribute weight vectors $W^{{\chi_{j} }} (j \in N)$ of supervisors, comprehensive PFM $G^{\chi } = \left[ {g_{ij}^{\chi } } \right]_{n \times m}$ of supervisors is constructed using Eq. (23). Then, satisfaction matrix $B = \left[ {b_{ij} } \right]_{m \times n}$ of supervisors for graduate students is constructed using Eqs. (24)-(28).

*Step 11* Based on satisfaction matrices $A = \left[ {a_{ij} } \right]_{m \times n}$ and $B = \left[ {b_{ij} } \right]_{m \times n}$ of GSS, matching matrix $X = \left[ {x_{ij} } \right]_{m \times n}$ and stable matching matrix $E = \left[ {\sigma_{ij}^{\Upsilon } } \right]_{m \times n}$ are introduced, and a many-to-one GSSM model (M-3) is constructed;

*Step 12* Extended satisfaction matrices $\tilde{A} = \left[ {\tilde{a}_{ig} } \right]_{m \times d}$ and $\tilde{B} = \left[ {\tilde{b}_{ig} } \right]_{m \times r}$ of graduate students and virtual supervisors are set up by introducing virtual supervisor subjects. Then model (M-3) is transformed into a one-to-one matching GSSM model (M-4).

*Step 13* The multi-objective matching model (M-4) is transformed into a single objective matching model (M-5) using linear weighting method; then model (M-5) is solved to obtain the optimal matching scheme $\Upsilon^{ * }$ between GSS.

## Example analysis

**Fig. 8:**
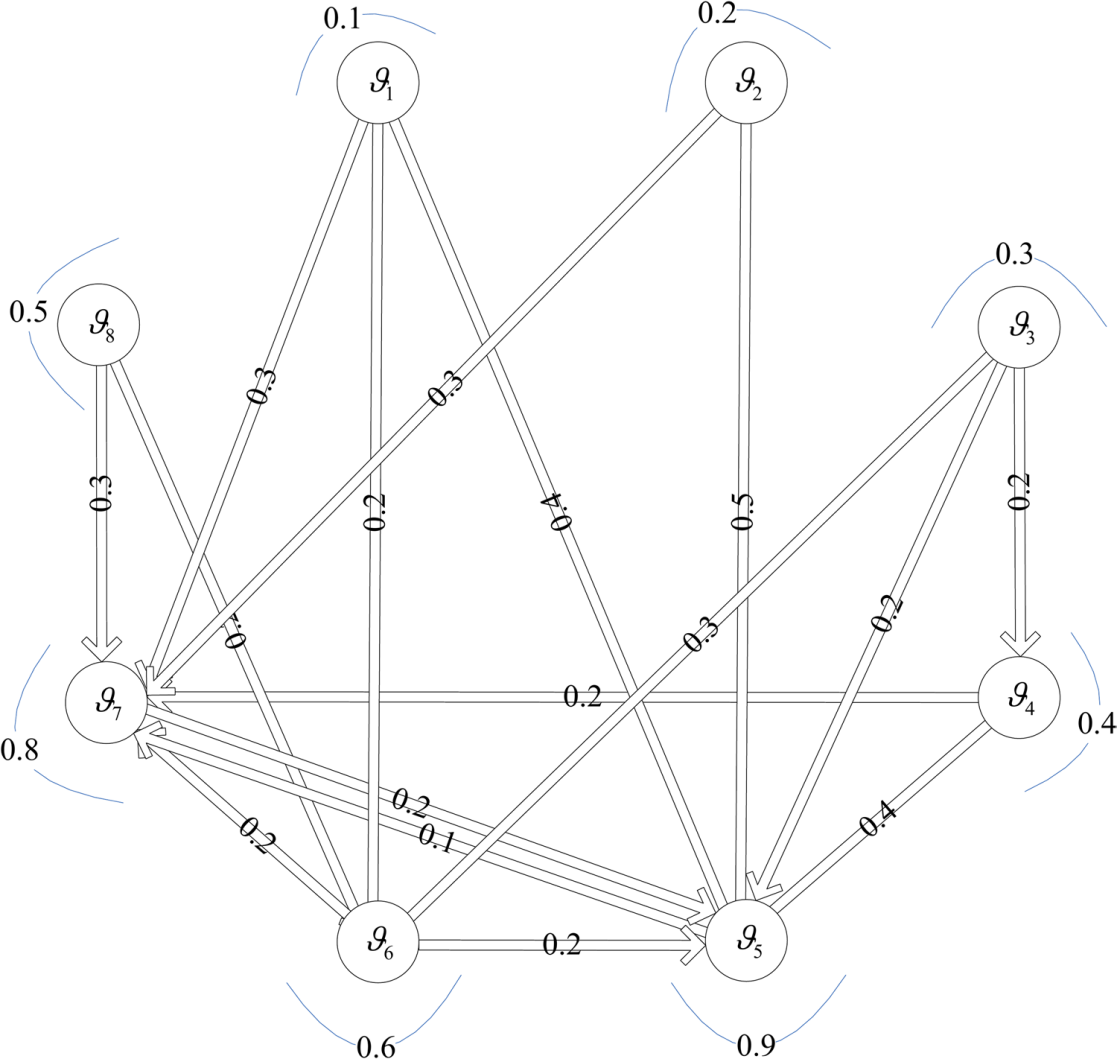
Transference relation of graduate students.

Consider the bilateral matching problem between graduate students majoring in Chinese Language and Literature and supervisors at a university in Shanghai. In 2023, there are 4 supervisors in Chinese Language and Literature major at the university; and the supervisor set is denoted as $\chi = \left\{ {\chi_{1} ,\chi_{2} ,\chi_{3} ,\chi_{4} } \right\}$. Among them, supervisor $\chi_{3}$ only admits one graduate student, supervisor $\chi_{2}$ admits three graduate students, and supervisors $\chi_{1}$ and $\chi_{4}$ admit two graduate students, namely $c_{3} = 1,1 \le e_{j} \le 3,j = 1,3,4$. This major has admitted 8 graduate students, and the graduate student set is denoted as $\vartheta = \left\{ {\vartheta_{1} ,\vartheta_{2} ,\vartheta_{3} ,\vartheta_{4} ,\vartheta_{5} ,\vartheta_{6} ,\vartheta_{7} ,\vartheta_{8} } \right\}$. Each graduate student can only match one supervisor. Supervisors evaluates graduate students based on four attributes: academic performance $\gamma_{1}^{\vartheta }$, English proficiency $\gamma_{2}^{\vartheta }$, academic experience $\gamma_{3}^{\vartheta }$ and learning autonomy $\gamma_{4}^{\vartheta }$; and the attribute set is denoted as $\gamma^{\vartheta } = \left\{ {\gamma_{1}^{\vartheta } ,\gamma_{2}^{\vartheta } ,\gamma_{3}^{\vartheta } ,\gamma_{4}^{\vartheta } } \right\}$. Graduate students evaluate supervisors based on four attributes: professional title $\gamma_{1}^{\chi }$, scholarly output $\gamma_{2}^{\chi }$, personal style $\gamma_{3}^{\chi }$ and research direction $\gamma_{4}^{\chi }$, and the attribute set is denoted as $\gamma^{\chi } = \left\{ {\gamma_{1}^{\chi } ,\gamma_{2}^{\chi } ,\gamma_{3}^{\chi } ,\gamma_{4}^{\chi } } \right\}$. GSS respectively use the linguistic term set $S = \left\{ {s_{0} ,s_{1} , \ldots ,s_{6} } \right\}$ to provide linguistic term matrices $S^{\vartheta } = \left[ {s_{itj}^{\vartheta } } \right]_{8 \times 4 \times 4}$ and $S^{\chi } = \left[ {s_{jli}^{\chi } } \right]_{4 \times 4 \times 8}$, as shown in Tables 3 and 4 respectively. Among them, $s_{0} =$ ‘extremely less consistent’, $s_{1} =$ ‘does not consistent’, $s_{2} =$ ‘less consistent’, $s_{3} =$ is ‘neutral’, $s_{4} =$ is ‘consistent’, $s_{5} =$ is ‘more consistent’, and $s_{6} =$ is ‘extremely consistent’. Graduate students provide transference relation, as shown in Fig. 8. Finally, the matching decision-making is made by the dean of the college.

**Table 3:** LTM $S^{\vartheta } = \left[ {s_{itj}^{\vartheta } } \right]_{8 \times 4 \times 4}$ of graduate students for supervisors.

	$\chi_{1}$				$\chi_{2}$				$\chi_{3}$				$\chi_{4}$			
	$\gamma_{1}^{\vartheta }$	$\gamma_{2}^{\vartheta }$	$\gamma_{3}^{\vartheta }$	$\gamma_{4}^{\vartheta }$	$\gamma_{1}^{\vartheta }$	$\gamma_{2}^{\vartheta }$	$\gamma_{3}^{\vartheta }$	$\gamma_{4}^{\vartheta }$	$\gamma_{1}^{\vartheta }$	$\gamma_{2}^{\vartheta }$	$\gamma_{3}^{\vartheta }$	$\gamma_{4}^{\vartheta }$	$\gamma_{1}^{\vartheta }$	$\gamma_{2}^{\vartheta }$	$\gamma_{3}^{\vartheta }$	$\gamma_{4}^{\vartheta }$
$\vartheta_{1}$	$s_{5}$	$s_{5}$	$s_{2}$	$s_{3}$	$s_{6}$	$s_{4}$	$s_{3}$	$s_{6}$	$s_{2}$	$s_{6}$	$s_{4}$	$s_{5}$	$s_{3}$	$s_{4}$	$s_{5}$	$s_{4}$
$\vartheta_{2}$	$s_{1}$	$s_{5}$	$s_{6}$	$s_{2}$	$s_{4}$	$s_{4}$	$s_{3}$	$s_{4}$	$s_{5}$	$s_{6}$	$s_{2}$	$s_{4}$	$s_{2}$	$s_{5}$	$s_{3}$	$s_{4}$
$\vartheta_{3}$	$s_{4}$	$s_{3}$	$s_{2}$	$s_{6}$	$s_{3}$	$s_{5}$	$s_{2}$	$s_{3}$	$s_{4}$	$s_{5}$	$s_{3}$	$s_{5}$	$s_{1}$	$s_{4}$	$s_{2}$	$s_{5}$
$\vartheta_{4}$	$s_{4}$	$s_{5}$	$s_{2}$	$s_{6}$	$s_{4}$	$s_{6}$	$s_{5}$	$s_{4}$	$s_{4}$	$s_{4}$	$s_{6}$	$s_{1}$	$s_{4}$	$s_{6}$	$s_{3}$	$s_{2}$
$\vartheta_{5}$	$s_{5}$	$s_{4}$	$s_{5}$	$s_{4}$	$s_{5}$	$s_{3}$	$s_{4}$	$s_{5}$	$s_{6}$	$s_{4}$	$s_{2}$	$s_{6}$	$s_{2}$	$s_{3}$	$s_{4}$	$s_{6}$
$\vartheta_{6}$	$s_{6}$	$s_{3}$	$s_{4}$	$s_{5}$	$s_{2}$	$s_{5}$	$s_{5}$	$s_{2}$	$s_{5}$	$s_{3}$	$s_{5}$	$s_{3}$	$s_{4}$	$s_{4}$	$s_{6}$	$s_{3}$
$\vartheta_{7}$	$s_{3}$	$s_{6}$	$s_{5}$	$s_{3}$	$s_{4}$	$s_{4}$	$s_{6}$	$s_{4}$	$s_{5}$	$s_{5}$	$s_{2}$	$s_{5}$	$s_{5}$	$s_{5}$	$s_{3}$	$s_{5}$
$\vartheta_{8}$	$s_{2}$	$s_{2}$	$s_{6}$	$s_{5}$	$s_{5}$	$s_{2}$	$s_{3}$	$s_{5}$	$s_{4}$	$s_{4}$	$s_{6}$	$s_{3}$	$s_{2}$	$s_{5}$	$s_{6}$	$s_{4}$

**Table 4:** LTM $S^{\chi } = \left[ {s_{jli}^{\chi } } \right]_{4 \times 4 \times 8}$ of supervisors for graduate students.

	$\vartheta_{1}$				$\vartheta_{2}$				$\vartheta_{3}$				$\vartheta_{4}$			
	$\gamma_{1}^{\chi }$	$\gamma_{2}^{\chi }$	$\gamma_{3}^{\chi }$	$\gamma_{4}^{\chi }$	$\gamma_{1}^{\chi }$	$\gamma_{2}^{\chi }$	$\gamma_{3}^{\chi }$	$\gamma_{4}^{\chi }$	$\gamma_{1}^{\chi }$	$\gamma_{2}^{\chi }$	$\gamma_{3}^{\chi }$	$\gamma_{4}^{\chi }$	$\gamma_{1}^{\chi }$	$\gamma_{2}^{\chi }$	$\gamma_{3}^{\chi }$	$\gamma_{4}^{\chi }$
$\chi_{1}$	$s_{3}$	$s_{4}$	$s_{2}$	$s_{4}$	$s_{6}$	$s_{5}$	$s_{2}$	$s_{2}$	$s_{6}$	$s_{1}$	$s_{5}$	$s_{0}$	$s_{3}$	$s_{3}$	$s_{2}$	$s_{6}$
$\chi_{2}$	$s_{0}$	$s_{6}$	$s_{0}$	$s_{1}$	$s_{6}$	$s_{2}$	$s_{3}$	$s_{2}$	$s_{3}$	$s_{5}$	$s_{5}$	$s_{2}$	$s_{5}$	$s_{2}$	$s_{5}$	$s_{3}$
$\chi_{3}$	$s_{0}$	$s_{4}$	$s_{4}$	$s_{5}$	$s_{2}$	$s_{5}$	$s_{1}$	$s_{2}$	$s_{4}$	$s_{0}$	$s_{5}$	$s_{3}$	$s_{2}$	$s_{1}$	$s_{4}$	$s_{1}$
$\chi_{4}$	$s_{3}$	$s_{5}$	$s_{3}$	$s_{4}$	$s_{4}$	$s_{2}$	$s_{3}$	$s_{3}$	$s_{6}$	$s_{4}$	$s_{3}$	$s_{4}$	$s_{5}$	$s_{4}$	$s_{5}$	$s_{1}$

	$\vartheta_{5}$				$\vartheta_{6}$				$\vartheta_{7}$				$\vartheta_{8}$			
	$\gamma_{1}^{\chi }$	$\gamma_{2}^{\chi }$	$\gamma_{3}^{\chi }$	$\gamma_{4}^{\chi }$	$\gamma_{1}^{\chi }$	$\gamma_{2}^{\chi }$	$\gamma_{3}^{\chi }$	$\gamma_{4}^{\chi }$	$\gamma_{1}^{\chi }$	$\gamma_{2}^{\chi }$	$\gamma_{3}^{\chi }$	$\gamma_{4}^{\chi }$	$\gamma_{1}^{\chi }$	$\gamma_{2}^{\chi }$	$\gamma_{3}^{\chi }$	$\gamma_{4}^{\chi }$
$\chi_{1}$	$s_{5}$	$s_{0}$	$s_{3}$	$s_{2}$	$s_{0}$	$s_{2}$	$s_{2}$	$s_{4}$	$s_{1}$	$s_{1}$	$s_{5}$	$s_{4}$	$s_{3}$	$s_{2}$	$s_{3}$	$s_{5}$
$\chi_{2}$	$s_{2}$	$s_{5}$	$s_{3}$	$s_{0}$	$s_{0}$	$s_{3}$	$s_{5}$	$s_{5}$	$s_{2}$	$s_{3}$	$s_{5}$	$s_{1}$	$s_{3}$	$s_{2}$	$s_{1}$	$s_{2}$
$\chi_{3}$	$s_{2}$	$s_{3}$	$s_{3}$	$s_{1}$	$s_{0}$	$s_{5}$	$s_{4}$	$s_{4}$	$s_{3}$	$s_{4}$	$s_{4}$	$s_{1}$	$s_{1}$	$s_{4}$	$s_{2}$	$s_{2}$
$\chi_{4}$	$s_{4}$	$s_{4}$	$s_{3}$	$s_{2}$	$s_{6}$	$s_{5}$	$s_{1}$	$s_{2}$	$s_{3}$	$s_{4}$	$s_{6}$	$s_{2}$	$s_{5}$	$s_{6}$	$s_{1}$	$s_{2}$

The method proposed in this paper is used to solve the above problem, and the solving process is as follows:

### Solution process

*Step 1* Based on LTM $S^{\vartheta } = \left[ {s_{itj}^{\vartheta } } \right]_{8 \times 4 \times 4}$, PFM $\Psi^{\vartheta } = \left[ {\varphi_{itj}^{\vartheta } } \right]_{8 \times 4 \times 4}$ of graduate students is constructed using Eq. (5).

*Step 2* Based on transference relation of graduate students, conformity coefficient matrix $V = \left[ {\wp_{ik} } \right]_{8 \times 8}$ of graduate students is constructed using Eqs. (7)-(9), as shown in Table 5, where $e = r = {1 \mathord{\left/ {\vphantom {1 2}} \right. \kern-0pt} 2}$.

**Table 5:** Conformity coefficient matrix $V = \left[ {\wp_{ik} } \right]_{8 \times 8}$ of graduate students.

	$\vartheta_{1}$	$\vartheta_{2}$	$\vartheta_{3}$	$\vartheta_{4}$	$\vartheta_{5}$	$\vartheta_{6}$	$\vartheta_{7}$	$\vartheta_{8}$
$\vartheta_{1}$	0.01	0.00	0.00	0.05	0.47	0.18	0.30	0.00
$\vartheta_{2}$	0.00	0.02	0.00	0.05	0.48	0.16	0.30	0.00
$\vartheta_{3}$	0.00	0.00	0.03	0.06	0.45	0.19	0.27	0.00
$\vartheta_{4}$	0.00	0.00	0.00	0.08	0.47	0.16	0.29	0.00
$\vartheta_{5}$	0.00	0.00	0.00	0.05	0.51	0.16	0.28	0.00
$\vartheta_{6}$	0.00	0.00	0.00	0.05	0.45	0.21	0.29	0.00
$\vartheta_{7}$	0.00	0.00	0.00	0.05	0.45	0.16	0.35	0.00
$\vartheta_{8}$	0.00	0.00	0.00	0.05	0.43	0.18	0.30	0.05

*Step 3* Based on PFM $\Psi^{\vartheta } = \left[ {\varphi_{itj}^{\vartheta } } \right]_{8 \times 4 \times 4}$ and conformity coefficient matrix $V = \left[ {\wp_{ik} } \right]_{8 \times 8}$ of graduate students, PFM $\Phi^{\vartheta } = \left[ {\phi_{itj}^{\vartheta } } \right]_{8 \times 4 \times 4}$ considering conformity psychology is constructed using Eq. (10), as shown in Table 6, where $\xi = 0.5$.

**Table 6:** PFM $\Phi^{\vartheta } = \left[ {\phi_{itj}^{\vartheta } } \right]_{8 \times 4 \times 4}$ considering conformity psychology.

	$\chi_{1}$				$\chi_{2}$			
	$\gamma_{1}^{\vartheta }$	$\gamma_{2}^{\vartheta }$	$\gamma_{3}^{\vartheta }$	$\gamma_{4}^{\vartheta }$	$\gamma_{1}^{\vartheta }$	$\gamma_{2}^{\vartheta }$	$\gamma_{3}^{\vartheta }$	$\gamma_{4}^{\vartheta }$
$\vartheta_{1}$	(0.76,0.53)	(0.75,0.56)	(0.55,0.61)	(0.56,0.53)	(0.8,0)	(0.62,0.54)	(0.62,0.57)	(0.79,0)
$\vartheta_{2}$	(0.51,0.7)	(0.76,0.57)	(0.86,0)	(0.5,0.63)	(0.65,0.55)	(0.62,0.54)	(0.62,0.57)	(0.8,0)
$\vartheta_{3}$	(0.68,0.56)	(0.59,0.57)	(0.55,0.62)	(0.79,0)	(0.57,0.53)	(0.71,0.52)	(0.55,0)	(0.64,0.54)
$\vartheta_{4}$	(0.68,0.57)	(0.76,0.58)	(0.56,0.62)	(0.79,0)	(0.66,0.56)	(0.77,0)	(0.79,0.58)	(0.52,0.62)
$\vartheta_{5}$	(0.81,0.63)	(0.74,0.69)	(0.82,0.6)	(0.71,0.63)	(0.78,0.62)	(0.65,0.61)	(0.77,0.69)	(0.77,0.6)
$\vartheta_{6}$	(0.84,0)	(0.63,0.61)	(0.72,0.57)	(0.73,0.55)	(0.56,0.66)	(0.72,0.55)	(0.8,0.6)	(0.67,0.57)
$\vartheta_{7}$	(0.67,0.59)	(0.84,0)	(0.81,0.56)	(0.63,0.58)	(0.7,0.61)	(0.67,0.6)	(0.87,0)	(0.64,0.57)
$\vartheta_{8}$	(0.54,0.65)	(0.53,0.69)	(0.86,0)	(0.72,0.52)	(0.73,0.53)	(0.5,0.63)	(0.62,0.57)	(0.73,0.51)

	$\chi_{3}$				$\chi_{4}$			
	$\gamma_{1}^{\vartheta }$	$\gamma_{2}^{\vartheta }$	$\gamma_{3}^{\vartheta }$	$\gamma_{4}^{\vartheta }$	$\gamma_{1}^{\vartheta }$	$\gamma_{2}^{\vartheta }$	$\gamma_{3}^{\vartheta }$	$\gamma_{4}^{\vartheta }$
$\vartheta_{1}$	(0.59,0.71)	(0.8,0)	(0.55,0.62)	(0.8,0.59)	(0.53,0.55)	(0.63,0.54)	(0.71,0.54)	(0.7,0.62)
$\vartheta_{2}$	(0.84,0.59)	(0.8,0)	(0.44,0.71)	(0.71,0.63)	(0.47,0.67)	(0.71,0.51)	(0.56,0.54)	(0.71,0.63)
$\vartheta_{3}$	(0.74,0.62)	(0.73,0.51)	(0.5,0.59)	(0.79,0.59)	(0.45,0.74)	(0.63,0.54)	(0.51,0.66)	(0.79,0.59)
$\vartheta_{4}$	(0.75,0.63)	(0.65,0.55)	(0.68,0)	(0.55,0.8)	(0.6,0.59)	(0.78,0)	(0.57,0.56)	(0.58,0.73)
$\vartheta_{5}$	(0.93,0)	(0.72,0.63)	(0.55,0.77)	(0.88,0)	(0.59,0.73)	(0.66,0.61)	(0.71,0.66)	(0.89,0)
$\vartheta_{6}$	(0.86,0.62)	(0.61,0.55)	(0.64,0.62)	(0.67,0.64)	(0.62,0.61)	(0.66,0.57)	(0.79,0)	(0.67,0.63)
$\vartheta_{7}$	(0.87,0.65)	(0.76,0.57)	(0.51,0.75)	(0.83,0)	(0.7,0.61)	(0.74,0.57)	(0.63,0.61)	(0.83,0.66)
$\vartheta_{8}$	(0.74,0.62)	(0.65,0.54)	(0.69,0)	(0.63,0.6)	(0.48,0.67)	(0.72,0.51)	(0.79,0)	(0.7,0.62)

*Note 4* If $\gamma_{3}^{\vartheta }$ is the cost attribute, then the PFM $\Phi^{\vartheta } = \left[ {\phi_{itj}^{\vartheta } } \right]_{8 \times 4 \times 4}$ (displayed in Table 6) can be transferred into BPF matrix $\Phi^{\prime \vartheta } = \left[ {\phi_{itj}^{\prime \vartheta } } \right]_{8 \times 4 \times 4}$ by Eq. (11), as shown in Table 7.

**Table 7:** BPF matrix $G^{\vartheta } = \left[ {g_{ij}^{\vartheta } } \right]_{8 \times 4}$ of graduate students for supervisors.

	$\chi_{1}$				$\chi_{2}$			
	$\gamma_{1}^{\vartheta }$	$\gamma_{2}^{\vartheta }$	$\gamma_{3}^{\vartheta }$	$\gamma_{4}^{\vartheta }$	$\gamma_{1}^{\vartheta }$	$\gamma_{2}^{\vartheta }$	$\gamma_{3}^{\vartheta }$	$\gamma_{4}^{\vartheta }$
$\vartheta_{1}$	(0.76,0.53)	(0.75,0.56)	(0.61,0.55)	(0.56,0.53)	(0.8,0)	(0.62,0.54)	(0.57,0.62)	(0.79,0)
$\vartheta_{2}$	(0.51,0.7)	(0.76,0.57)	(0,0.86)	(0.5,0.63)	(0.65,0.55)	(0.62,0.54)	(0.57,0.62)	(0.8,0)
$\vartheta_{3}$	(0.68,0.56)	(0.59,0.57)	(0.62,0.55)	(0.79,0)	(0.57,0.53)	(0.71,0.52)	(0,0.55)	(0.64,0.54)
$\vartheta_{4}$	(0.68,0.57)	(0.76,0.58)	(0.62,0.56)	(0.79,0)	(0.66,0.56)	(0.77,0)	(0.58,0.79)	(0.52,0.62)
$\vartheta_{5}$	(0.81,0.63)	(0.74,0.69)	(0.6,0.82)	(0.71,0.63)	(0.78,0.62)	(0.65,0.61)	(0.69,0.77)	(0.77,0.6)
$\vartheta_{6}$	(0.84,0)	(0.63,0.61)	(0.57,0.72)	(0.73,0.55)	(0.56,0.66)	(0.72,0.55)	(0.6,0.8)	(0.67,0.57)
$\vartheta_{7}$	(0.67,0.59)	(0.84,0)	(0.56,0.81)	(0.63,0.58)	(0.7,0.61)	(0.67,0.6)	(0,0.87)	(0.64,0.57)
$\vartheta_{8}$	(0.54,0.65)	(0.53,0.69)	(0,0.86)	(0.72,0.52)	(0.73,0.53)	(0.5,0.63)	(0.57,0.62)	(0.73,0.51)

	$\chi_{3}$				$\chi_{4}$			
	$\gamma_{1}^{\vartheta }$	$\gamma_{2}^{\vartheta }$	$\gamma_{3}^{\vartheta }$	$\gamma_{4}^{\vartheta }$	$\gamma_{1}^{\vartheta }$	$\gamma_{2}^{\vartheta }$	$\gamma_{3}^{\vartheta }$	$\gamma_{4}^{\vartheta }$
$\vartheta_{1}$	(0.59,0.71)	(0.8,0)	(0.62,0.55)	(0.8,0.59)	(0.53,0.55)	(0.63,0.54)	(0.54,0.71)	(0.7,0.62)
$\vartheta_{2}$	(0.84,0.59)	(0.8,0)	(0.71,0.44)	(0.71,0.63)	(0.47,0.67)	(0.71,0.51)	(0.54,0.56)	(0.71,0.63)
$\vartheta_{3}$	(0.74,0.62)	(0.73,0.51)	(0.59,0.5)	(0.79,0.59)	(0.45,0.74)	(0.63,0.54)	(0.66,0.51)	(0.79,0.59)
$\vartheta_{4}$	(0.75,0.63)	(0.65,0.55)	(0,0.68)	(0.55,0.8)	(0.6,0.59)	(0.78,0)	(0.56,0.57)	(0.58,0.73)
$\vartheta_{5}$	(0.93,0)	(0.72,0.63)	(0.77,0.55)	(0.88,0)	(0.59,0.73)	(0.66,0.61)	(0.66,0.71)	(0.89,0)
$\vartheta_{6}$	(0.86,0.62)	(0.61,0.55)	(0.62,0.64)	(0.67,0.64)	(0.62,0.61)	(0.66,0.57)	(0,0.79)	(0.67,0.63)
$\vartheta_{7}$	(0.87,0.65)	(0.76,0.57)	(0.75,0.51)	(0.83,0)	(0.7,0.61)	(0.74,0.57)	(0.61,0.63)	(0.83,0.66)
$\vartheta_{8}$	(0.74,0.62)	(0.65,0.54)	(0,0.69)	(0.63,0.6)	(0.48,0.67)	(0.72,0.51)	(0,0.79)	(0.7,0.62)

*Step 4* Based on attribute comparison information provided by graduate students and conformity coefficient matrix $V = \left[ {\wp_{ik} } \right]_{8 \times 8}$, attribute weight vectors $W^{{\vartheta_{1} }} = \left( {0.23,0.17,0.09,0.51} \right)$, $W^{{\vartheta_{2} }} = \left( {0.16,0.28,0.12,0.43} \right)$, $W^{{\vartheta_{3} }} = \left( {0.38,0.21,0.09,0.32} \right)$, $W^{{\vartheta_{4} }} = \left( {0.32,0.24,0.22,0.22} \right)$, $W^{{\vartheta_{5} }} = \left( {0.17,0.26,0.15,0.42} \right)$, $W^{{\vartheta_{6} }} = \left( {0.37,0.28,0.17,0.18} \right)$, $W^{{\vartheta_{7} }} = \left( {0.25,0.23,0.14,0.39} \right)$, $W^{{\vartheta_{8} }} = \left( {0.33,0.29,0.17,0.21} \right)$ of graduate students are calculated using Algorithm 2.

*Step 5* Based on PFM $\Phi^{\vartheta } = \left[ {\phi_{itj}^{\vartheta } } \right]_{8 \times 4 \times 4}$ and attribute weight vectors $W^{{\vartheta_{i} }} (i = 1,2, \ldots ,8)$, comprehensive PFM $G^{\vartheta } = \left[ {g_{ij}^{\vartheta } } \right]_{8 \times 4}$ of graduate students is built using Eq. (13), as shown in Table 8. Based on comprehensive PFM $G^{\vartheta } = \left[ {g_{ij}^{\vartheta } } \right]_{8 \times 4}$, positive ideal solution $g_{i}^{ + } =${(0.74, 0.37), (0.75, 0.14), (0.8, 0.28), (0.75, 0.38)} and negative ideal solution $g_{i}^{ - } =${(0.64, 0.55), (0.64, 0.56), (0.67, 0.51), (0.60, 0.64)} of graduate students are obtained. Then satisfaction matrix $A = \left[ {a_{ij} } \right]_{8 \times 4}$ of graduate students for supervisors is constructed using Eqs. (14)-(18), as shown in Table 9, where $\tau = 0.5$.

**Table 8:** Comprehensive PFM $G^{\vartheta } = \left[ {g_{ij}^{\vartheta } } \right]_{8 \times 4}$ of graduate students.

	$\chi_{1}$	$\chi_{2}$	$\chi_{3}$	$\chi_{4}$
$\vartheta_{1}$	(0.64, 0.54)	(0.75, 0.14)	(0.73, 0.52)	(0.65, 0.58)
$\vartheta_{2}$	(0.62, 0.55)	(0.70, 0.31)	(0.72, 0.45)	(0.65, 0.59)
$\vartheta_{3}$	(0.68, 0.39)	(0.62, 0.48)	(0.73, 0.59)	(0.60, 0.64)
$\vartheta_{4}$	(0.70, 0.46)	(0.68, 0.45)	(0.67, 0.51)	(0.63, 0.47)
$\vartheta_{5}$	(0.75, 0.64)	(0.74, 0.62)	(0.80, 0.28)	(0.75, 0.38)
$\vartheta_{6}$	(0.74, 0.37)	(0.66, 0.60)	(0.72, 0.60)	(0.67, 0.50)
$\vartheta_{7}$	(0.71, 0.45)	(0.69, 0.51)	(0.78, 0.39)	(0.75, 0.62)
$\vartheta_{8}$	(0.63, 0.53)	(0.64, 0.56)	(0.68, 0.49)	(0.65, 0.50)

**Table 9:** Satisfaction matrix $A = \left[ {a_{ij} } \right]_{8 \times 4}$ of graduate students for supervisors.

	$\chi_{1}$	$\chi_{2}$	$\chi_{3}$	$\chi_{4}$
$\vartheta_{1}$	0.27	0.88	0.38	0.35
$\vartheta_{2}$	0.17	0.69	0.42	0.33
$\vartheta_{3}$	0.61	0.40	0.43	0.14
$\vartheta_{4}$	0.57	0.44	0.16	0.54
$\vartheta_{5}$	0.52	0.40	0.85	0.87
$\vartheta_{6}$	0.84	0.26	0.42	0.57
$\vartheta_{7}$	0.61	0.29	0.64	0.48
$\vartheta_{8}$	0.26	0.14	0.25	0.54

*Step 6* Based on LTM $S^{\chi } = \left[ {s_{jli}^{\chi } } \right]_{4 \times 4 \times 8}$, PFM $\Psi^{\chi } = \left[ {\varphi_{jli}^{\chi } } \right]_{4 \times 4 \times 8}$ of supervisors is constructed using Eq. (5).

*Step 7* Based on comprehensive PFM $G^{\vartheta } = \left[ {g_{ij}^{\vartheta } } \right]_{8 \times 4}$, preference coefficient matrix $U = \left[ {\mu_{ij}^{\vartheta } } \right]_{8 \times 4}$ of graduate students is constructed using Eqs. (19)-(21), as shown in Table 10, where $\theta = 2.25$.

**Table 10:** Preference coefficient $U = \left[ {\mu_{ij}^{\vartheta } } \right]_{8 \times 4}$ of graduate students.

	$\chi_{1}$	$\chi_{2}$	$\chi_{3}$	$\chi_{4}$
$\vartheta_{1}$	0.13	1.00	0.33	0.15
$\vartheta_{2}$	0.07	0.77	0.43	0.13
$\vartheta_{3}$	0.59	0.21	0.28	0.03
$\vartheta_{4}$	0.46	0.50	0.19	0.34
$\vartheta_{5}$	0.40	0.30	0.82	0.83
$\vartheta_{6}$	0.69	0.00	0.03	0.37
$\vartheta_{7}$	0.54	0.37	0.63	0.82
$\vartheta_{8}$	0.10	0.08	0.21	0.34

*Step 8* Based on PFM $\Psi^{\chi } = \left[ {\varphi_{jli}^{\chi } } \right]_{4 \times 4 \times 8}$ and preference coefficient matrix $U = \left[ {\mu_{ij}^{\vartheta } } \right]_{8 \times 4}$, PFM $\Phi^{\chi } = \left[ {\phi_{jli}^{\chi } } \right]_{4 \times 4 \times 8}$ considering graduate students’ preferences is set up using Eq. (22), as shown in Table 11, where $\beta = 0.5$.

**Table 11:** PFM $\Phi^{\chi } = \left[ {\phi_{jli}^{\chi } } \right]_{4 \times 4 \times 8}$ considering graduate students’ preferences.

	$\vartheta_{1}$				$\vartheta_{2}$			
	$\gamma_{1}^{\chi }$	$\gamma_{2}^{\chi }$	$\gamma_{3}^{\chi }$	$\gamma_{4}^{\chi }$	$\gamma_{1}^{\chi }$	$\gamma_{2}^{\chi }$	$\gamma_{3}^{\chi }$	$\gamma_{4}^{\chi }$
$\chi_{1}$	(0.72,0.77)	(0.55,0.78)	(0.6,0.66)	(0.74,0.74)	(0.75,0)	(0.56,0.79)	(0.61,0.67)	(0.74,0.76)
$\chi_{2}$	(0,0.81)	(0.65,0)	(0,0.67)	(0.3,0.76)	(0.78,0)	(0.47,0.68)	(0.59,0.52)	(0.35,0.71)
$\chi_{3}$	(0,0.76)	(0.65,0.6)	(0.59,0.61)	(0.51,0.72)	(0.49,0.71)	(0.67,0.57)	(0.51,0.65)	(0.44,0.75)
$\chi_{4}$	(0.69,0.62)	(0.73,0.63)	(0.63,0.72)	(0.49,0.75)	(0.71,0.62)	(0.7,0.65)	(0.64,0.72)	(0.49,0.75)

	$\vartheta_{3}$				$\vartheta_{4}$			
	$\gamma_{1}^{\chi }$	$\gamma_{2}^{\chi }$	$\gamma_{3}^{\chi }$	$\gamma_{4}^{\chi }$	$\gamma_{1}^{\chi }$	$\gamma_{2}^{\chi }$	$\gamma_{3}^{\chi }$	$\gamma_{4}^{\chi }$
$\chi_{1}$	(0.75,0)	(0.42,0.78)	(0.6,0.55)	(0,0.76)	(0.64,0.69)	(0.48,0.69)	(0.51,0.63)	(0.76,0)
$\chi_{2}$	(0.73,0.76)	(0.65,0.71)	(0.75,0.63)	(0.46,0.78)	(0.75,0.68)	(0.53,0.71)	(0.73,0.57)	(0.43,0.68)
$\chi_{3}$	(0.56,0.7)	(0,0.66)	(0.62,0.61)	(0.48,0.73)	(0.54,0.74)	(0.64,0.66)	(0.61,0.64)	(0.48,0.8)
$\chi_{4}$	(0.73,0)	(0.74,0.66)	(0.66,0.75)	(0.51,0.78)	(0.7,0.58)	(0.69,0.6)	(0.64,0.67)	(0.43,0.77)

	$\vartheta_{5}$				$\vartheta_{6}$			
	$\gamma_{1}^{\chi }$	$\gamma_{2}^{\chi }$	$\gamma_{3}^{\chi }$	$\gamma_{4}^{\chi }$	$\gamma_{1}^{\chi }$	$\gamma_{2}^{\chi }$	$\gamma_{3}^{\chi }$	$\gamma_{4}^{\chi }$
$\chi_{1}$	(0.72,0.7)	(0,0.8)	(0.55,0.59)	(0.63,0.72)	(0,0.8)	(0.41,0.72)	(0.46,0.6)	(0.65,0.63)
$\chi_{2}$	(0.69,0.78)	(0.64,0.69)	(0.69,0.61)	(0,0.8)	(0,0.82)	(0.66,0.77)	(0.77,0.68)	(0.51,0.8)
$\chi_{3}$	(0.41,0.67)	(0.53,0.5)	(0.48,0.51)	(0.35,0.77)	(0,0.76)	(0.7,0.66)	(0.63,0.67)	(0.53,0.8)
$\chi_{4}$	(0.6,0.51)	(0.62,0.52)	(0.5,0.57)	(0.35,0.69)	(0.72,0)	(0.72,0.58)	(0.55,0.74)	(0.43,0.74)

	$\vartheta_{7}$				$\vartheta_{8}$			
	$\gamma_{1}^{\chi }$	$\gamma_{2}^{\chi }$	$\gamma_{3}^{\chi }$	$\gamma_{4}^{\chi }$	$\gamma_{1}^{\chi }$	$\gamma_{2}^{\chi }$	$\gamma_{3}^{\chi }$	$\gamma_{4}^{\chi }$
$\chi_{1}$	(0.57,0.78)	(0.43,0.78)	(0.6,0.56)	(0.67,0.65)	(0.73,0.78)	(0.54,0.8)	(0.61,0.65)	(0.75,0.74)
$\chi_{2}$	(0.67,0.77)	(0.58,0.68)	(0.74,0.59)	(0.42,0.79)	(0.77,0.79)	(0.64,0.76)	(0.74,0.67)	(0.49,0.79)
$\chi_{3}$	(0.48,0.61)	(0.61,0.55)	(0.55,0.56)	(0.39,0.78)	(0.53,0.75)	(0.67,0.62)	(0.58,0.65)	(0.48,0.78)
$\chi_{4}$	(0.55,0.49)	(0.62,0.52)	(0.66,0)	(0.35,0.69)	(0.7,0.58)	(0.74,0)	(0.56,0.74)	(0.44,0.74)

*Step 9* Based on attribute comparison information provided by supervisors, attribute weight vectors $W^{{\chi_{1} }} = \left( {0.14,0.05,0.58,0.23} \right)$, $W^{{\chi_{2} }} = \left( {0.44,0.17,0.29,0.10} \right)$, $W^{{\chi_{3} }} = \left( {0.42,0.25,0.18,0.14} \right)$ and $W^{{\chi_{4} }} = \left( {0.58,0.23,0.14,0.05} \right)$ of supervisors are determined using Algorithm 5.

*Step 10* Based on PFM $\Phi^{\chi } = \left[ {\phi_{jli}^{\chi } } \right]_{4 \times 4 \times 8}$ and attribute weight vectors $W^{{\chi_{j} }} (j = 1,2,3,4)$, comprehensive PFM $G^{\chi } = \left[ {g_{ij}^{\chi } } \right]_{8 \times 4}$ of supervisors is built using Eq. (23), as shown in Table 12. Based on comprehensive PFM $G^{\chi } = \left[ {g_{ij}^{\chi } } \right]_{8 \times 4}$, the positive ideal solution $g_{j}^{ + } =${(0.68, 0.64), (0.63, 0.34), (0.71, 0.3), (0.68, 0.6), (0.58, 0.53), (0.68, 0.28), (0.68, 0.47)}, the negative ideal solution $g_{j}^{ - } =${(0.35, 0.69), (0.53, 0.67), (0.42, 0.68), (0.57, 0.71), (0.61, 0.72), (0.39, 0.77), (0.51, 0.61), (0.57, 0.7)} of supervisors are obtained. Satisfaction matrix $B = \left[ {b_{ij} } \right]_{8 \times 4}$ of supervisors for graduate students is set up using Eqs. (24)-(28), as shown in Table 13, where $\tau = 0.5$.

**Table 12:** Comprehensive PFM $G^{\chi } = \left[ {g_{ij}^{\chi } } \right]_{8 \times 4}$ of supervisors.

	$\chi_{1}$	$\chi_{2}$	$\chi_{3}$	$\chi_{4}$
$\vartheta_{1}$	(0.65, 0.7)	(0.14, 0.62)	(0.35, 0.69)	(0.68, 0.64)
$\vartheta_{2}$	(0.66, 0.6)	(0.63, 0.34)	(0.53, 0.67)	(0.68, 0.65)
$\vartheta_{3}$	(0.47, 0.53)	(0.7, 0.72)	(0.42, 0.68)	(0.71, 0.3)
$\vartheta_{4}$	(0.59, 0.49)	(0.67, 0.65)	(0.57, 0.71)	(0.68, 0.6)
$\vartheta_{5}$	(0.57, 0.65)	(0.61, 0.72)	(0.44, 0.61)	(0.58, 0.53)
$\vartheta_{6}$	(0.44, 0.64)	(0.39, 0.77)	(0.37, 0.72)	(0.68, 0.28)
$\vartheta_{7}$	(0.6, 0.62)	(0.65, 0.7)	(0.51, 0.61)	(0.57, 0.44)
$\vartheta_{8}$	(0.66, 0.7)	(0.71, 0.75)	(0.57, 0.7)	(0.68, 0.47)

**Table 13:** Satisfaction Matrix $B = \left[ {b_{ij} } \right]_{8 \times 4}$ of supervisors for graduate students.

	$\chi_{1}$	$\chi_{2}$	$\chi_{3}$	$\chi_{4}$
$\vartheta_{1}$	0.71	0.33	0.15	0.85
$\vartheta_{2}$	0.39	0.85	0.15	0.37
$\vartheta_{3}$	0.42	0.47	0.14	0.86
$\vartheta_{4}$	0.51	0.61	0.20	0.80
$\vartheta_{5}$	0.51	0.16	0.65	0.84
$\vartheta_{6}$	0.38	0.13	0.24	0.88
$\vartheta_{7}$	0.38	0.43	0.19	0.81
$\vartheta_{8}$	0.35	0.42	0.16	0.84

*Step 11* Based on satisfaction matrices $A = \left[ {a_{ij} } \right]_{8 \times 4}$ and $B = \left[ {b_{ij} } \right]_{8 \times 4}$ of GSS, and the introduced matching matrix $X = \left[ {x_{ij} } \right]_{8 \times 4}$ and stable matching matrix $E = \left[ {\sigma_{ij}^{\Upsilon } } \right]_{8 \times 4}$, many-to-one GSSM model (M-3) is constructed. Then, model (M-3) is transformed into a one-to-one matching GSSM model (M-4) by introducing virtual supervisor subjects.

*Step 12* Model (M-4) is transformed into the single objective model (M-5) using linear weighting method. Then, model (M-5) is solved to obtain the optimal matching matrix $\Theta = \left[ \alpha \right]_{8 \times 8}$, as shown in Table 14. According to Table 14, the optimal matching scheme between GSS is $\Upsilon_{1}^{ * } = \left\{ {\left( {\vartheta_{1} ,\chi_{2} } \right),\left( {\vartheta_{2} ,\chi_{2} } \right),\left( {\vartheta_{3} ,\chi_{1} } \right),\left( {\vartheta_{4} ,\chi_{2} } \right),\left( {\vartheta_{5} ,\chi_{3} } \right),\left( {\vartheta_{6} ,\chi_{4} } \right),\left( {\vartheta_{7} ,\chi_{1} } \right),\left( {\vartheta_{8} ,\chi_{4} } \right)} \right\}$, where $\omega_{1} = \omega_{2} = \omega_{3} = {1 \mathord{\left/ {\vphantom {1 3}} \right. \kern-0pt} 3}$.

**Table 14:** Matching Matrix $\Theta = \left[ \alpha \right]_{8 \times 8}$.

	$\chi_{11}$	$\chi_{12}$	$\chi_{23}$	$\chi_{24}$	$\chi_{25}$	$\chi_{36}$	$\chi_{47}$	$\chi_{48}$
$\vartheta_{1}$	0	0	1	0	0	0	0	0
$\vartheta_{2}$	0	0	0	0	1	0	0	0
$\vartheta_{3}$	1	0	0	0	0	0	0	0
$\vartheta_{4}$	0	0	0	1	0	0	0	0
$\vartheta_{5}$	0	0	0	0	0	1	0	0
$\vartheta_{6}$	0	0	0	0	0	0	0	1
$\vartheta_{7}$	0	1	0	0	0	0	0	0
$\vartheta_{8}$	0	0	0	0	0	0	1	0

### Sensitivity analysis

This paper involves the values of several parameters, including grey correlation coefficient $\tau$ and weight vector $\Omega = \left( {\omega_{1} ,\omega_{2} ,\omega_{3} } \right)$ of the objective function. Sensitivity analysis will be conducted on the above parameters.

**Fig. 9:**
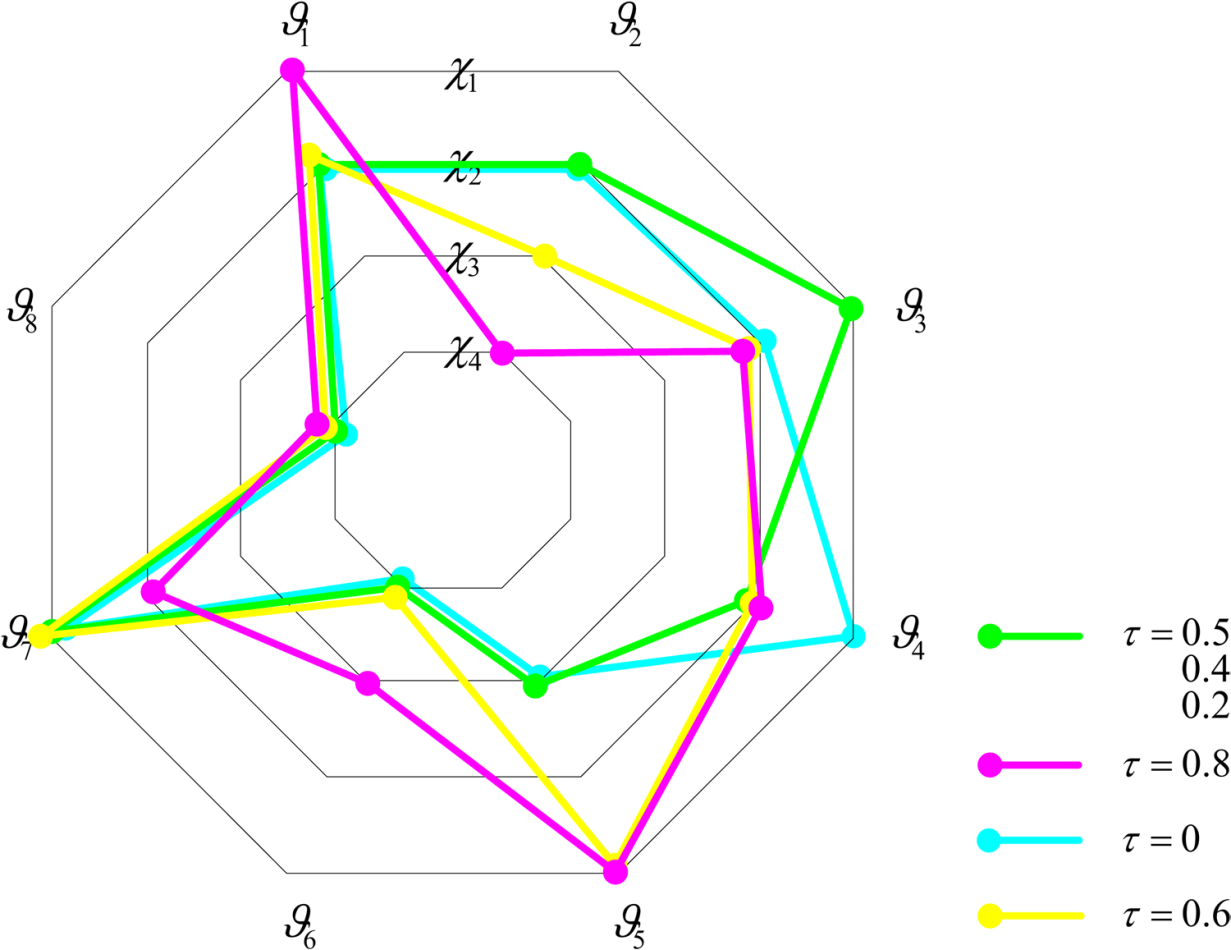
Optimal matching scheme $\Upsilon^{ * }$ based on grey correlation coefficient $\tau$.

**Fig. 10:**
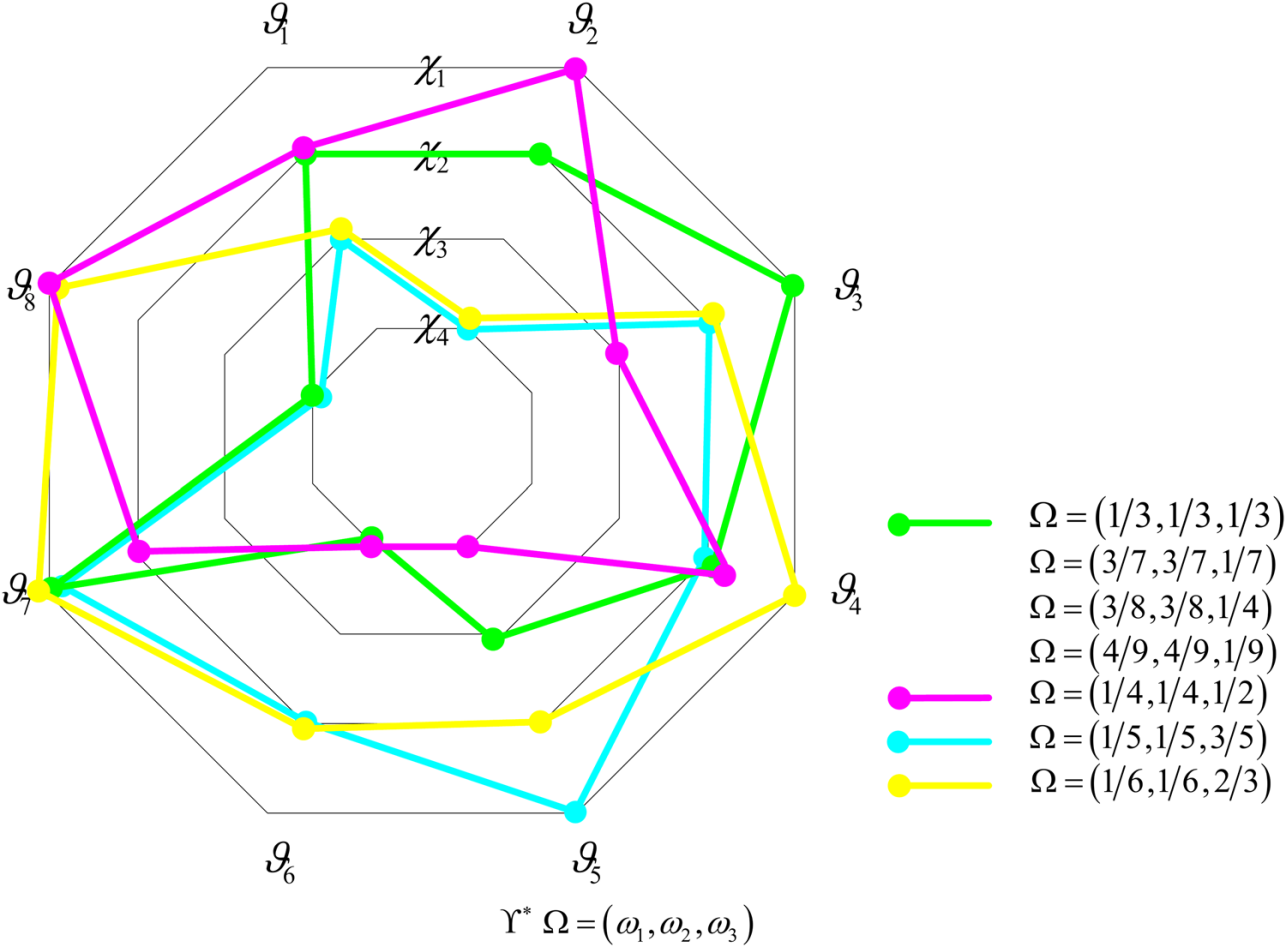
Optimal matching scheme $\Upsilon^{ * }$ based on weight vector $\Omega = \left( {\omega_{1} ,\omega_{2} ,\omega_{3} } \right)$.

1. The grey correlation coefficient $\tau$ is used to adjust the proportion of gray correlation degrees and closeness in the calculation of satisfaction. When grey correlation coefficient $\tau$ is set to 0.8, 0.6, 0.5, 0.4, 0.2 and 0, the optimal matching schemes $\Upsilon_{1}^{ * } - \Upsilon_{4}^{ * }$ for GSS are obtained, as shown in Table 15 and Fig. 9. It can be inferred that there are certain differences in the optimal matching schemes obtained using different grey correlation coefficients. Therefore, the value of grey correlation coefficient $\tau$ should be fully considered when solving the above problem.
2. When the weight vector $\Omega = \left( {\omega_{1} ,\omega_{2} ,\omega_{3} } \right)$ is assigned values of $\left( {{1 \mathord{\left/ {\vphantom {1 3}} \right. \kern-0pt} 3},{1 \mathord{\left/ {\vphantom {1 3}} \right. \kern-0pt} 3},{1 \mathord{\left/ {\vphantom {1 3}} \right. \kern-0pt} 3}} \right)$, $\left( {{1 \mathord{\left/ {\vphantom {1 4}} \right. \kern-0pt} 4},{1 \mathord{\left/ {\vphantom {1 4}} \right. \kern-0pt} 4},{1 \mathord{\left/ {\vphantom {1 2}} \right. \kern-0pt} 2}} \right)$, $\left( {{1 \mathord{\left/ {\vphantom {1 5}} \right. \kern-0pt} 5},{1 \mathord{\left/ {\vphantom {1 5}} \right. \kern-0pt} 5},{3 \mathord{\left/ {\vphantom {3 5}} \right. \kern-0pt} 5}} \right)$, $\left( {{1 \mathord{\left/ {\vphantom {1 6}} \right. \kern-0pt} 6},{1 \mathord{\left/ {\vphantom {1 6}} \right. \kern-0pt} 6},{2 \mathord{\left/ {\vphantom {2 3}} \right. \kern-0pt} 3}} \right)$, $\left( {{3 \mathord{\left/ {\vphantom {3 7}} \right. \kern-0pt} 7},{3 \mathord{\left/ {\vphantom {3 7}} \right. \kern-0pt} 7},{1 \mathord{\left/ {\vphantom {1 7}} \right. \kern-0pt} 7}} \right)$, $\left( {{3 \mathord{\left/ {\vphantom {3 8}} \right. \kern-0pt} 8},{3 \mathord{\left/ {\vphantom {3 8}} \right. \kern-0pt} 8},{1 \mathord{\left/ {\vphantom {1 4}} \right. \kern-0pt} 4}} \right)$, and $\left( {{4 \mathord{\left/ {\vphantom {4 9}} \right. \kern-0pt} 9},{4 \mathord{\left/ {\vphantom {4 9}} \right. \kern-0pt} 9},{1 \mathord{\left/ {\vphantom {1 9}} \right. \kern-0pt} 9}} \right)$, the optimal matching schemes for GSS as follow: $\Upsilon_{1}^{ * } ,\Upsilon_{5}^{ * } ,\Upsilon_{6}^{ * }$, and $\Upsilon_{7}^{ * }$, as demonstrated in Table 16 and Fig. 10. These results reveal significant discrepancies among the optimal schemes derived from different weight vectors. Consequently, the selection of $\Omega = \left( {\omega_{1} ,\omega_{2} ,\omega_{3} } \right)$ requires careful evaluation when solving the above problem.

**Table 15:** Optimal matching scheme $\Upsilon^{ * }$ based on grey correlation coefficient $\tau$.

Grey correlation coefficient $\tau$	Optimal matching scheme $\Upsilon^{ * }$
$\tau = 0.8$	$\Upsilon_{2}^{ * } = \left\{ {\left( {\vartheta_{1} ,\chi_{1} } \right),\left( {\vartheta_{2} ,\chi_{4} } \right),\left( {\vartheta_{3} ,\chi_{2} } \right),\left( {\vartheta_{4} ,\chi_{2} } \right),\left( {\vartheta_{5} ,\chi_{1} } \right),\left( {\vartheta_{6} ,\chi_{3} } \right),\left( {\vartheta_{7} ,\chi_{2} } \right),\left( {\vartheta_{8} ,\chi_{4} } \right)} \right\}$
$\tau = 0.6$	$\Upsilon_{3}^{ * } = \left\{ {\left( {\vartheta_{1} ,\chi_{2} } \right),\left( {\vartheta_{2} ,\chi_{2} } \right),\left( {\vartheta_{3} ,\chi_{2} } \right),\left( {\vartheta_{4} ,\chi_{1} } \right),\left( {\vartheta_{5} ,\chi_{3} } \right),\left( {\vartheta_{6} ,\chi_{4} } \right),\left( {\vartheta_{7} ,\chi_{1} } \right),\left( {\vartheta_{8} ,\chi_{4} } \right)} \right\}$
$\tau = 0.5$	$\Upsilon_{1}^{ * } = \left\{ {\left( {\vartheta_{1} ,\chi_{2} } \right),\left( {\vartheta_{2} ,\chi_{2} } \right),\left( {\vartheta_{3} ,\chi_{1} } \right),\left( {\vartheta_{4} ,\chi_{2} } \right),\left( {\vartheta_{5} ,\chi_{3} } \right),\left( {\vartheta_{6} ,\chi_{4} } \right),\left( {\vartheta_{7} ,\chi_{1} } \right),\left( {\vartheta_{8} ,\chi_{4} } \right)} \right\}$
$\tau = 0.4$	$\Upsilon_{1}^{ * } = \left\{ {\left( {\vartheta_{1} ,\chi_{2} } \right),\left( {\vartheta_{2} ,\chi_{2} } \right),\left( {\vartheta_{3} ,\chi_{1} } \right),\left( {\vartheta_{4} ,\chi_{2} } \right),\left( {\vartheta_{5} ,\chi_{3} } \right),\left( {\vartheta_{6} ,\chi_{4} } \right),\left( {\vartheta_{7} ,\chi_{1} } \right),\left( {\vartheta_{8} ,\chi_{4} } \right)} \right\}$
$\tau = 0.2$	$\Upsilon_{1}^{ * } = \left\{ {\left( {\vartheta_{1} ,\chi_{2} } \right),\left( {\vartheta_{2} ,\chi_{2} } \right),\left( {\vartheta_{3} ,\chi_{1} } \right),\left( {\vartheta_{4} ,\chi_{2} } \right),\left( {\vartheta_{5} ,\chi_{3} } \right),\left( {\vartheta_{6} ,\chi_{4} } \right),\left( {\vartheta_{7} ,\chi_{1} } \right),\left( {\vartheta_{8} ,\chi_{4} } \right)} \right\}$
$\tau = 0$	$\Upsilon_{4}^{ * } = \left\{ {\left( {\vartheta_{1} ,\chi_{2} } \right),\left( {\vartheta_{2} ,\chi_{3} } \right),\left( {\vartheta_{3} ,\chi_{2} } \right),\left( {\vartheta_{4} ,\chi_{2} } \right),\left( {\vartheta_{5} ,\chi_{1} } \right),\left( {\vartheta_{6} ,\chi_{4} } \right),\left( {\vartheta_{7} ,\chi_{1} } \right),\left( {\vartheta_{8} ,\chi_{4} } \right)} \right\}$

**Table 16:** Optimal matching scheme $\Upsilon^{ * }$ based on weight vector $\Omega = \left( {\omega_{1} ,\omega_{2} ,\omega_{3} } \right)$.

Weight vector $\Omega$	Optimal matching scheme $\Upsilon^{ * }$
$\Omega = \left( {{1 \mathord{\left/ {\vphantom {1 3}} \right. \kern-0pt} 3},{1 \mathord{\left/ {\vphantom {1 3}} \right. \kern-0pt} 3},{1 \mathord{\left/ {\vphantom {1 3}} \right. \kern-0pt} 3}} \right)$	$\Upsilon_{1}^{ * } = \left\{ {\left( {\vartheta_{1} ,\chi_{2} } \right),\left( {\vartheta_{2} ,\chi_{2} } \right),\left( {\vartheta_{3} ,\chi_{1} } \right),\left( {\vartheta_{4} ,\chi_{2} } \right),\left( {\vartheta_{5} ,\chi_{3} } \right),\left( {\vartheta_{6} ,\chi_{4} } \right),\left( {\vartheta_{7} ,\chi_{1} } \right),\left( {\vartheta_{8} ,\chi_{4} } \right)} \right\}$
$\Omega = \left( {{1 \mathord{\left/ {\vphantom {1 4}} \right. \kern-0pt} 4},{1 \mathord{\left/ {\vphantom {1 4}} \right. \kern-0pt} 4},{1 \mathord{\left/ {\vphantom {1 2}} \right. \kern-0pt} 2}} \right)$	$\Upsilon_{5}^{ * } = \left\{ {\left( {\vartheta_{1} ,\chi_{2} } \right),\left( {\vartheta_{2} ,\chi_{1} } \right),\left( {\vartheta_{3} ,\chi_{3} } \right),\left( {\vartheta_{4} ,\chi_{2} } \right),\left( {\vartheta_{5} ,\chi_{4} } \right),\left( {\vartheta_{6} ,\chi_{4} } \right),\left( {\vartheta_{7} ,\chi_{2} } \right),\left( {\vartheta_{8} ,\chi_{1} } \right)} \right\}$
$\Omega = \left( {{1 \mathord{\left/ {\vphantom {1 5}} \right. \kern-0pt} 5},{1 \mathord{\left/ {\vphantom {1 5}} \right. \kern-0pt} 5},{3 \mathord{\left/ {\vphantom {3 5}} \right. \kern-0pt} 5}} \right)$	$\Upsilon_{6}^{ * } = \left\{ {\left( {\vartheta_{1} ,\chi_{3} } \right),\left( {\vartheta_{2} ,\chi_{4} } \right),\left( {\vartheta_{3} ,\chi_{2} } \right),\left( {\vartheta_{4} ,\chi_{2} } \right),\left( {\vartheta_{5} ,\chi_{1} } \right),\left( {\vartheta_{6} ,\chi_{2} } \right),\left( {\vartheta_{7} ,\chi_{1} } \right),\left( {\vartheta_{8} ,\chi_{4} } \right)} \right\}$
$\Omega = \left( {{1 \mathord{\left/ {\vphantom {1 6}} \right. \kern-0pt} 6},{1 \mathord{\left/ {\vphantom {1 6}} \right. \kern-0pt} 6},{2 \mathord{\left/ {\vphantom {2 3}} \right. \kern-0pt} 3}} \right)$	$\Upsilon_{7}^{ * } = \left\{ {\left( {\vartheta_{1} ,\chi_{3} } \right),\left( {\vartheta_{2} ,\chi_{4} } \right),\left( {\vartheta_{3} ,\chi_{2} } \right),\left( {\vartheta_{4} ,\chi_{4} } \right),\left( {\vartheta_{5} ,\chi_{2} } \right),\left( {\vartheta_{6} ,\chi_{2} } \right),\left( {\vartheta_{7} ,\chi_{1} } \right),\left( {\vartheta_{8} ,\chi_{1} } \right)} \right\}$
$\Omega = \left( {{3 \mathord{\left/ {\vphantom {3 7}} \right. \kern-0pt} 7},{3 \mathord{\left/ {\vphantom {3 7}} \right. \kern-0pt} 7},{1 \mathord{\left/ {\vphantom {1 7}} \right. \kern-0pt} 7}} \right)$	$\Upsilon_{1}^{ * } = \left\{ {\left( {\vartheta_{1} ,\chi_{2} } \right),\left( {\vartheta_{2} ,\chi_{2} } \right),\left( {\vartheta_{3} ,\chi_{1} } \right),\left( {\vartheta_{4} ,\chi_{2} } \right),\left( {\vartheta_{5} ,\chi_{3} } \right),\left( {\vartheta_{6} ,\chi_{4} } \right),\left( {\vartheta_{7} ,\chi_{1} } \right),\left( {\vartheta_{8} ,\chi_{4} } \right)} \right\}$
$\Omega = \left( {{3 \mathord{\left/ {\vphantom {3 8}} \right. \kern-0pt} 8},{3 \mathord{\left/ {\vphantom {3 8}} \right. \kern-0pt} 8},{1 \mathord{\left/ {\vphantom {1 4}} \right. \kern-0pt} 4}} \right)$	$\Upsilon_{1}^{ * } = \left\{ {\left( {\vartheta_{1} ,\chi_{2} } \right),\left( {\vartheta_{2} ,\chi_{2} } \right),\left( {\vartheta_{3} ,\chi_{1} } \right),\left( {\vartheta_{4} ,\chi_{2} } \right),\left( {\vartheta_{5} ,\chi_{3} } \right),\left( {\vartheta_{6} ,\chi_{4} } \right),\left( {\vartheta_{7} ,\chi_{1} } \right),\left( {\vartheta_{8} ,\chi_{4} } \right)} \right\}$
$\Omega = \left( {{4 \mathord{\left/ {\vphantom {4 9}} \right. \kern-0pt} 9},{4 \mathord{\left/ {\vphantom {4 9}} \right. \kern-0pt} 9},{1 \mathord{\left/ {\vphantom {1 9}} \right. \kern-0pt} 9}} \right)$	$\Upsilon_{1}^{ * } = \left\{ {\left( {\vartheta_{1} ,\chi_{2} } \right),\left( {\vartheta_{2} ,\chi_{2} } \right),\left( {\vartheta_{3} ,\chi_{1} } \right),\left( {\vartheta_{4} ,\chi_{2} } \right),\left( {\vartheta_{5} ,\chi_{3} } \right),\left( {\vartheta_{6} ,\chi_{4} } \right),\left( {\vartheta_{7} ,\chi_{1} } \right),\left( {\vartheta_{8} ,\chi_{4} } \right)} \right\}$

### Comparative analysis

**Fig. 11:**
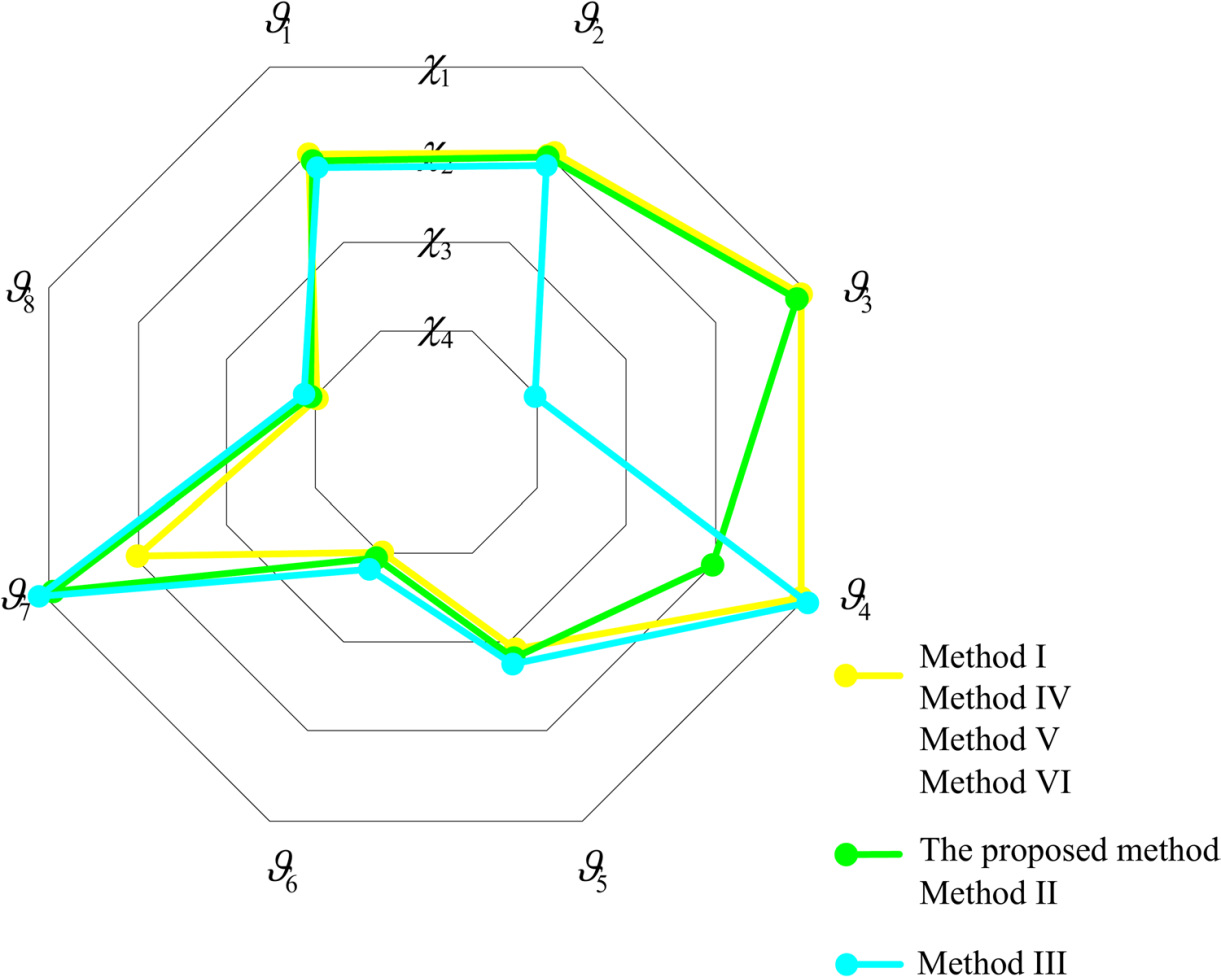
Comparative analysis based on different methods.

This subsection will verify the effectiveness and advantages of the proposed method by comparing it with the existing research. Methods proposed by Zhao et al.^53^, Zhang et al.^54^, Pu et al.^55^, Pandey et al.^56^ and Hu et al.^57^ have been adapted and extended; these are referred to as Method I to Method VI, respectively. The comparative optimal matching results derived from the proposed method and Method I to Method VI are shown in Table 17 and Fig. 11.

**Table 17:** Comparison results of different methods.

Method	Optimal matching scheme $\Upsilon^{ * }$
The proposed method	$\Upsilon_{1}^{ * } = \left\{ {\left( {\vartheta_{1} ,\chi_{2} } \right),\left( {\vartheta_{2} ,\chi_{2} } \right),\left( {\vartheta_{3} ,\chi_{1} } \right),\left( {\vartheta_{4} ,\chi_{2} } \right),\left( {\vartheta_{5} ,\chi_{3} } \right),\left( {\vartheta_{6} ,\chi_{4} } \right),\left( {\vartheta_{7} ,\chi_{1} } \right),\left( {\vartheta_{8} ,\chi_{4} } \right)} \right\}$
Method I	$\Upsilon_{7}^{ * } = \left\{ {\left( {\vartheta_{1} ,\chi_{2} } \right),\left( {\vartheta_{2} ,\chi_{2} } \right),\left( {\vartheta_{3} ,\chi_{1} } \right),\left( {\vartheta_{4} ,\chi_{1} } \right),\left( {\vartheta_{5} ,\chi_{3} } \right),\left( {\vartheta_{6} ,\chi_{4} } \right),\left( {\vartheta_{7} ,\chi_{2} } \right),\left( {\vartheta_{8} ,\chi_{4} } \right)} \right\}$
Method II	$\Upsilon_{1}^{ * } = \left\{ {\left( {\vartheta_{1} ,\chi_{2} } \right),\left( {\vartheta_{2} ,\chi_{2} } \right),\left( {\vartheta_{3} ,\chi_{1} } \right),\left( {\vartheta_{4} ,\chi_{2} } \right),\left( {\vartheta_{5} ,\chi_{3} } \right),\left( {\vartheta_{6} ,\chi_{4} } \right),\left( {\vartheta_{7} ,\chi_{1} } \right),\left( {\vartheta_{8} ,\chi_{4} } \right)} \right\}$
Method III	$\Upsilon_{8}^{ * } = \left\{ {\left( {\vartheta_{1} ,\chi_{2} } \right),\left( {\vartheta_{2} ,\chi_{2} } \right),\left( {\vartheta_{3} ,\chi_{2} } \right),\left( {\vartheta_{4} ,\chi_{1} } \right),\left( {\vartheta_{5} ,\chi_{3} } \right),\left( {\vartheta_{6} ,\chi_{4} } \right),\left( {\vartheta_{7} ,\chi_{1} } \right),\left( {\vartheta_{8} ,\chi_{4} } \right)} \right\}$
Method IV	$\Upsilon_{7}^{ * } = \left\{ {\left( {\vartheta_{1} ,\chi_{2} } \right),\left( {\vartheta_{2} ,\chi_{2} } \right),\left( {\vartheta_{3} ,\chi_{1} } \right),\left( {\vartheta_{4} ,\chi_{1} } \right),\left( {\vartheta_{5} ,\chi_{3} } \right),\left( {\vartheta_{6} ,\chi_{4} } \right),\left( {\vartheta_{7} ,\chi_{2} } \right),\left( {\vartheta_{8} ,\chi_{4} } \right)} \right\}$
Method V	$\Upsilon_{7}^{ * } = \left\{ {\left( {\vartheta_{1} ,\chi_{2} } \right),\left( {\vartheta_{2} ,\chi_{2} } \right),\left( {\vartheta_{3} ,\chi_{1} } \right),\left( {\vartheta_{4} ,\chi_{1} } \right),\left( {\vartheta_{5} ,\chi_{3} } \right),\left( {\vartheta_{6} ,\chi_{4} } \right),\left( {\vartheta_{7} ,\chi_{2} } \right),\left( {\vartheta_{8} ,\chi_{4} } \right)} \right\}$
Method VI	$\Upsilon_{7}^{ * } = \left\{ {\left( {\vartheta_{1} ,\chi_{2} } \right),\left( {\vartheta_{2} ,\chi_{2} } \right),\left( {\vartheta_{3} ,\chi_{1} } \right),\left( {\vartheta_{4} ,\chi_{1} } \right),\left( {\vartheta_{5} ,\chi_{3} } \right),\left( {\vartheta_{6} ,\chi_{4} } \right),\left( {\vartheta_{7} ,\chi_{2} } \right),\left( {\vartheta_{8} ,\chi_{4} } \right)} \right\}$

Table 17 and Fig. 11 reveal significant variations in the optimal matching schemes derived from different decision methods. Specifically, the optimal matching schemes obtained by Method I, Method IV, Method V, and Method VI are $\Upsilon_{7}^{ * }$; the ones obtained by the proposed method and Method II are $\Upsilon_{1}^{ * }$; the ones obtained by Method III is $\Upsilon_{8}^{ * }$. Main reasons for the differences are as follows: (1) Method I employs stability constraints to solve the above problem under goals of maximizing satisfactions of bilateral subjects, which ignores the matching fairness. (2) Methods II and III addressed the above issue under goals of maximizing satisfactions of bilateral subjects and minimization of satisfaction gaps without stability constraints. Among them, absolute value of satisfaction gaps is used to calculate the matching fairness in Method II; a new calculation method is designed and used to calculate the one in Method III. While they all ignore the matching stability. (3) Methods IV and V used stability constraints to solve the above problem under goals of maximizing satisfactions of bilateral subjects and minimization of satisfaction gaps. Among them, calculation of fairness in Methods IV and V references to Methods II and III respectively. (4) Method VI proposed a matching model combining bilateral satisfaction thresholds with stability constraints.

However, the proposed method distinguishes itself from Methods I-VI as follows: (1) The proposed method introduced a new concept of stability-based fairness. It takes into account both the matching fairness of bilateral subjects and the stability of matching scheme. (2) In terms of model design, Methods I–V set stability as the constraint and fairness as the third goal, which means the stability directly constrains all goals of the model. However, the proposed method incorporates stability-based fairness as the third goal, which means only the third goal is directly constrained by stability. (3) In coordinating decision-making criteria, method VI introduced bilateral satisfaction thresholds, which can control the lower limit of satisfaction for matching pairs. However, the proposed method not only considers stability, fairness, and satisfaction levels of bilateral subjects in the matching decision, but also provides a new idea for harmonizing fairness, stability and satisfaction levels of matching goals. These features demonstrate that stability-based fairness enhances both the practicality and effectiveness of real-world decision-making in bilateral matching contexts.

In summary, the advantages of the proposed method are reflected in satisfaction calculation and model construction respectively. In terms of satisfaction calculation, (1) the method for converting multi granularity LTSs to PFNs is expanded, which fully considering the fuzziness, uncertainty and hesitation of bilateral subjects’ preferences; (2) conformity psychology and graduate students’ preferences in GSSM are introduced to better describe subjects’ preferences and calculate attribute weights; (3) a more reasonable satisfaction calculation using TOPSIS and grey correlation degrees is provided. In terms of model construction, a new method is proposed to calculate fairness and stability matching by introducing the concept of stability-based fairness, then a GSSM model considering stability-based fairness is set up to obtain a stability and fairness matching scheme.

### Advantages and limitations of the proposed method

Based on the above analysis, advantages of the proposed method are as follows:

1. The method for converting multi granularity LTSs to PFNs is extended, explicitly accounting for the fuzziness, uncertainty and hesitation inherent in bilateral subjects’ preferences. This method enables subjects to express preferences more flexibly while preserving the integrity of their original preference information.
2. Conformity psychology and graduate students’ preferences are introduced to better describe subjects’ preferences and calculate attribute weights in GSSM, which addressing the impact of graduate students’ psychological tendencies on BMDM.
3. A hybrid satisfaction calculation method is developed using TOPSIS and grey correlation degrees, which can more reasonably reflect satisfactions of GSS.
4. A novel GSSM model considering stability-based fairness is set up to obtain a stability and more fairness matching scheme. Therefore, the designed model is more suitable for GSSM decision-making, which helps to improve the effectiveness of decision-making.

The limitations of the proposed method are dispalyed as follows: (1) The method for converting multi granularity LTSs into PFNs is preliminarily discussed in this paper, but only the one-way conversion is studied. In addition, conversion theories between other LTSs and PFNs are not further explored. (2) The impact of graduate students’ preferences on supervisors’ decision-making is researched, but the impact of supervisors’ preferences on graduate students’ decision-making is not discussed. (3) Only the impact of conformity psychology on BMDM is studied. Other psychological behaviors and the cross effects of multiple psychological behaviors are not studied.

### Managerial implications of this paper

Management implications includes both theoretical and practical implications. Theoretical implications are displayed in the following aspects: (1) The decision-making method for GSSM considering stability-based fairness is proposed, which considers the fairness and stability of matching process and satisfactions of bilateral subjects. (2) The proposed many-to-one matching method considering the influence of conformity psychology and individual preferences is explored, which is more conducive to matching graduate students with the most suitable supervisors and avoiding conflicts. (3) In the actual decision-making process, LTSs are usually used to collect preference information of bilateral subjects due to their characteristics of easy to understand. Furthermore, LTSs are transformed into PFNs, which makes it easier to obtained decision information while preserving the uncertainty of preference information. (4) A method for calculating graduate students’ preference coefficients based on TODIM is proposed, which introduced a calculation for individual preferences. (5) Satisfaction calculation methods are designed based on grey correlation degrees and TOPSIS, which consider the conformity psychology and preferences of graduate students. (6) A many-to-one GSSM model considering stability-based fairness is established, which reflects the fairness and stability of matching.

Practical implications are reflected in the following aspects: (1) A novel method considering stability-based fairness is proposed to solve the GSSM problem. It helps universities make GSSM decisions, improves the efficiency of resource allocation for GSS, and enhance the quality of graduate education in universities. (2) The psychology of the special group of graduate students, including conformity psychology, loss aversion and fairness psychology, is taken into account the GSSM process. It helps to reduce potential conflicts between GSS during the matching process and improve their satisfactions.

## Conclusions

This paper explores the influence of graduate students’ conformity psychology and preferences on GSSM decision-making, and proposes a many-to-one matching method considering stability-based fairness. First, conformity coefficients of graduate students are calculated to construct the PFM considering conformity psychology and attribute weight vectors of graduate students. Second, the preference coefficient matrix of graduate students is built; then the PFM considering graduate students’ preferences and attribute weight vectors of supervisors are determined. On this basis, satisfaction matrices of GSS are set up using TOPSIS and grey correlation degrees. Furthermore, a many-to-one GSSM model considering stability-based fairness is established. The one-to-one GSSM model is set up by introducing virtual supervisor subjects. The above model is solved to obtain the optimal matching scheme between GSS.

Compared with the existing methods, the method proposed in this paper has the following significant features: (1) The conversion method for multi granularity LTSs to PFNs is provided. (2) An improved score of PFNs is proposed. (3) Methods for adjusting initial preferences of graduate students using conformity coefficients and adjust initial preferences of supervisors using graduate students’ preferences are developed. (4) The weight calculation method that combines conformity psychology with BWM is studied. (5) Satisfaction using TOPSIS and grey correlation degrees is calculated. (6) A GSSM model that considers stability-based fairness is established.

Limitations of this paper are as follows: (1) This paper preliminarily discusses the method for converting multi granularity LTSs to PFNs. (2) The impact of supervisors’ preferences on graduate student decision-making are not studied. (3) Only the influence of conformity psychology on BMDM is considered in this paper.

In view of the above limitations, the following potential research directions are pointed out: (1) The conversion theory of other complex linguistic preferences should be developed. (2) The influence of supervisors’ preferences on graduate students decision-making should be studied. (3) Cross effects of different psychological behaviors of bilateral subjects on BMDM should be researched.

## Data Availability

All data generated or analyzed during this study are included in this article.
